# Mapping Polyclonal HIV-1 Antibody Responses via Next-Generation Neutralization Fingerprinting

**DOI:** 10.1371/journal.ppat.1006148

**Published:** 2017-01-04

**Authors:** Nicole A. Doria-Rose, Han R. Altae-Tran, Ryan S. Roark, Stephen D. Schmidt, Matthew S. Sutton, Mark K. Louder, Gwo-Yu Chuang, Robert T. Bailer, Valerie Cortez, Rui Kong, Krisha McKee, Sijy O’Dell, Felicia Wang, Salim S. Abdool Karim, James M. Binley, Mark Connors, Barton F. Haynes, Malcolm A. Martin, David C. Montefiori, Lynn Morris, Julie Overbaugh, Peter D. Kwong, John R. Mascola, Ivelin S. Georgiev

**Affiliations:** 1 Vaccine Research Center, National Institutes of Health, Bethesda, MD, United States of America; 2 Human Biology Division, Fred Hutchinson Cancer Research Center, Seattle, WA, United States of America; 3 Program in Molecular and Cellular Biology, University of Washington, Seattle, WA, United States of America; 4 Vanderbilt Vaccine Center, Vanderbilt University, Nashville, TN, United States of America; 5 Centre for the AIDS Programme of Research in South Africa (CAPRISA), University of KwaZulu-Natal, Durban, South Africa; 6 Department of Epidemiology, Columbia University, New York, NY, United States of America; 7 San Diego Biomedical Research Institute, San Diego, CA, United States of America; 8 HIV-Specific Immunity Section, National Institutes of Health, Bethesda, MD, United States of America; 9 Duke University Human Vaccine Institute, Durham, NC, United States of America; 10 Departments of Medicine and Immunology, Duke University School of Medicine, Durham, NC, United States of America; 11 Center for HIV/AIDS Vaccine Immunology-Immunogen Discovery at Duke University, Durham, NC, United States of America; 12 Laboratory of Molecular Microbiology, National Institute of Allergy and Infectious Diseases, National Institutes of Health, Bethesda, MD, United States of America; 13 Department of Surgery, Duke University School of Medicine, Durham, NC, United States of America; 14 University of the Witwatersrand, Johannesburg, South Africa; 15 Center for HIV and STIs, National Institute for Communicable Diseases, Johannesburg, South Africa; 16 Department of Pathology, Microbiology, and Immunology, Vanderbilt University Medical Center, Nashville, TN, United States of America; 17 Department of Electrical Engineering and Computer Science, Vanderbilt University, Nashville, TN, United States of America; University of Zurich, SWITZERLAND

## Abstract

Computational neutralization fingerprinting, NFP, is an efficient and accurate method for predicting the epitope specificities of polyclonal antibody responses to HIV-1 infection. Here, we present next-generation NFP algorithms that substantially improve prediction accuracy for individual donors and enable serologic analysis for entire cohorts. Specifically, we developed algorithms for: (a) selection of optimized virus neutralization panels for NFP analysis, (b) estimation of NFP prediction confidence for each serum sample, and (c) identification of sera with potentially novel epitope specificities. At the individual donor level, the next-generation NFP algorithms particularly improved the ability to detect multiple epitope specificities in a sample, as confirmed both for computationally simulated polyclonal sera and for samples from HIV-infected donors. Specifically, the next-generation NFP algorithms detected multiple specificities in twice as many samples of simulated sera. Further, unlike the first-generation NFP, the new algorithms were able to detect both of the previously confirmed antibody specificities, VRC01-like and PG9-like, in donor CHAVI 0219. At the cohort level, analysis of ~150 broadly neutralizing HIV-infected donor samples suggested a potential connection between clade of infection and types of elicited epitope specificities. Most notably, while 10E8-like antibodies were observed in infections from different clades, an enrichment of such antibodies was predicted for clade B samples. Ultimately, such large-scale analyses of antibody responses to HIV-1 infection can help guide the design of epitope-specific vaccines that are tailored to take into account the prevalence of infecting clades within a specific geographic region. Overall, the next-generation NFP technology will be an important tool for the analysis of broadly neutralizing polyclonal antibody responses against HIV-1.

## Author Summary

HIV-1 remains a significant global health threat, with no effective vaccine against the virus currently available. Since traditional vaccine design efforts have had limited success, much effort in recent years has focused on gaining a better understanding of the ways select individuals are able to effectively neutralize the virus upon natural infection, and to utilize that knowledge for the design of optimized vaccine candidates. Primary emphasis has been placed on characterizing the antibody arm of the immune system, and specifically on antibodies capable of neutralizing the majority of circulating HIV-1 strains. Various experimental techniques can be applied to map the epitope targets of these antibodies, but more recently, the development of computational methods has provided an efficient and accurate alternative for understanding the complex antibody responses to HIV-1 in a given individual. Here, we present the next generation of this computational technology, and show that these new methods have significantly improved accuracy and confidence, and that they enable the interrogation of biologically important questions that can lead to new insights for the design of an effective vaccine against HIV-1.

## Introduction

The HIV-1 Env glycoprotein, the sole target of antibody responses on the surface of the virus, exhibits extreme levels of sequence diversity [1–3], possibly explaining why antibodies capable of broad and potent neutralization of the virus have been found to target only a small set of conserved Env sites of vulnerability [1, 4]. A large number of broadly neutralizing HIV-1 antibodies (bNAbs) have been isolated within the last decade [5–20] and have been shown to be useful in preclinical studies of therapy and prevention [21–30]. Yet, no HIV-1 vaccine capable of eliciting such bNAbs is currently available. With the limited success of traditional vaccinology approaches, significant effort has been devoted to “rational” vaccine design based on understanding and manipulating the interactions between bNAbs and HIV-1 [31–34]. The identification and characterization of antibodies from infected or vaccinated individuals provides insights into the specifics of the antibody response against the virus [35–47] and can help generate templates for antibody-specific vaccine design [48, 49]. A challenge to the field is that neutralizing antibody responses to HIV-1 infection or vaccination are complex and difficult to deconvolute, often comprised of diverse bNAb lineages targeting a variety of epitopes on Env [4, 12, 18, 50]. Mapping the epitope specificities of polyclonal HIV-1 antibody responses therefore requires substantial effort. Standard epitope mapping methods include experimental techniques such as binding competition with monoclonal antibodies, neutralization or binding of Env variants containing epitope-specific knockout mutations, and neutralization blocking by epitope-specific antigens [9, 50–57]. These methods often fail to yield definitive answers, particularly when more than one specificity is targeted by the serum, or when the true epitope target has not yet been defined.

In addition to these experimental methods, mapping of antibody responses can be achieved through computational analysis of the neutralization of diverse HIV-1 strains by serum or plasma [4, 58]. Previously, we developed and validated the NFP (neutralization fingerprinting) algorithm for delineating antibody specificities in polyclonal sera [4]. The NFP algorithm uses a reference set of monoclonal antibody neutralization fingerprints (the potency pattern with which an antibody neutralizes a set of HIV-1 strains) in order to estimate the relative contribution of different types of known antibody specificities to the neutralization by a given polyclonal serum [4] (S1A Fig). Since serum neutralization data is typically obtained in the very first steps of serum characterization, algorithms like NFP can be substantially more efficient, and may also present advantages and improved ability for detecting antibody specificities in polyclonal sera, compared to standard methods [4]. NFP has been successfully used for mapping the antibody specificities in previously uncharacterized sera, for selecting donors for the isolation of broad and potent HIV-1 neutralizing antibodies, and for analysis of the dominant antibody specificities found during different stages of infection [1, 4, 12, 14, 35, 59].

**Fig 1:**
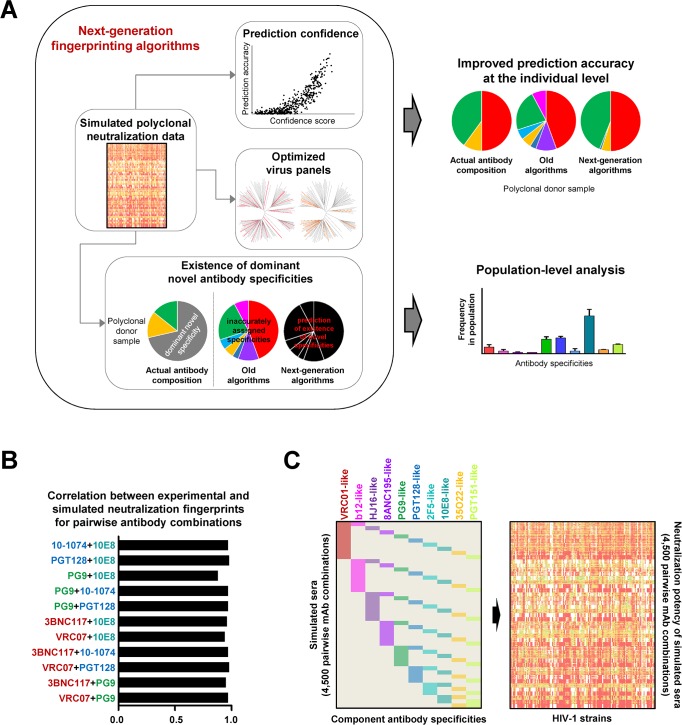
Next-generation neutralization fingerprinting algorithms for analysis of polyclonal responses against HIV-1. (A) Schematic of algorithm advances and applications. Large-scale simulations of polyclonal neutralization data were used as a benchmark for the development of the different algorithm components: algorithms for improved confidence in the computational predictions, for selection of optimized virus panels, and for prediction of potentially novel antibody specificities in a serum neutralization signal. Together, these algorithms build the foundation for improving the NFP prediction accuracy at the individual level and enabling NFP analysis at the population (or cohort) level. (B) Spearman correlation coefficients for simulated vs. experimentally determined neutralization data for combinations of antibody pairs. Antibody names are colored according to the respective epitope-specific clusters from (C). (C) Large-scale simulation of polyclonal antibody neutralization, represented as a heatmap based on neutralization potency (white-green-yellow-red).

Here, we present next-generation serum mapping algorithms that address some of the major challenges for the NFP approach, substantially improving the accuracy of the computational predictions and enabling the exploration of new biological questions (Figs 1A and S1B). Specifically, the next-generation NFP approach involves the following new algorithm developments (S1B Fig): First, we developed a method for simulating HIV-1 neutralization by polyclonal antibodies in order to circumvent the limited availability of serum neutralization data from donors with well-defined specificities. Using the simulated neutralization as a quantitative benchmark for algorithm performance, we further developed algorithms for optimizing the selection of HIV-1 strains for virus neutralization assays, as well as for identifying sera with potentially novel antibody specificities and for estimating the confidence in the computational predictions for a given serum. The significance of these new NFP algorithm capabilities was confirmed through analysis of both simulated and real donor sera. Finally, extending from the analysis at the individual donor level, we developed algorithms that specifically enable polyclonal antibody analysis for entire cohorts. To confirm the utility of the next-generation NFP algorithms, we performed cohort-level analysis of a diverse set of HIV-infected donors. Our analysis suggested a potential connection between plasma clade and types of elicited antibody specificities, and therefore has direct implications for the design of epitope-specific HIV-1 vaccines. Overall, the next-generation NFP technology provides a valuable resource for analysis of broadly neutralizing antibody responses to HIV-1 infection.

## Results

### Generation of simulated sera

The ability to compare and evaluate the performance of computational algorithms requires the development of quantitative benchmarks. In the case of NFP, success is determined by the ability of the algorithms to delineate component antibody specificities in polyclonal sera. Real donor sera, however, are not suitable as quantitative benchmarks because the actual antibody composition of a given polyclonal serum cannot be fully defined with current technologies, even for sera with extensively characterized antibody specificities. An alternative solution would be to utilize mixtures (or combinations) of monoclonal antibodies that can, to an approximation, serve as a proxy for polyclonal serum analysis. First, we analyzed published data for combinations of 2, 3, and 4 bNAbs with different epitope specificities [60], and applied the NFP algorithm to delineate the component antibody specificities from the bNAb combination data (S2A Fig). For all but one of the 21 combinations, including the two 4-bNAb combinations, the top-scoring NFP predictions matched the actual component antibody specificities, suggesting that such monoclonal combination data could be used as a benchmark for the NFP algorithm optimization. Since experimental data for bNAb combinations is limited, we developed a method for large-scale simulation of polyclonal neutralization data (Figs 1B and 1C and S2B and S2C). Specifically, simulated sera were generated as pairwise bNAb combinations that mixed equal amounts of an antibody representative from two of the ten epitope-specific antibody groups (see Materials and Methods). The simulation method showed excellent agreement with experimental data for a set of 11 pairwise bNAb combinations, with Spearman correlations between simulated and experimentally determined neutralization data ranging from 0.88 to 0.98 (median of 0.97) (Figs 1B and S2B). Given these results, we proceeded to generate large-scale sets of simulated neutralization data for different pairwise bNAb combinations, to be used as a quantitative benchmark for NFP algorithm evaluation (Figs 1C and S2C).

### Identification of HIV-1 virus panels for improved NFP accuracy

#### Computational search for improved virus panels

Antibody neutralization data against hundreds of HIV-1 viral variants is available for a large number of monoclonal antibodies [7, 9, 61, 62]. While serum neutralization data of such magnitude may also be possible to obtain [63], in many cases limited serum sample availability necessitates the use of substantially smaller virus panels [64]. In our previous work, the NFP algorithm utilized a panel of 21 diverse HIV-1 strains for which both monoclonal antibody and polyclonal serum neutralization data were available, as a proof of principle for neutralization-based serum mapping [1, 4, 12, 35]. While this 21-strain panel has been successfully applied in a number of cases, we set out to explore whether optimizing the composition and size of the virus panel can further improve NFP prediction accuracy.

**Fig 2:**
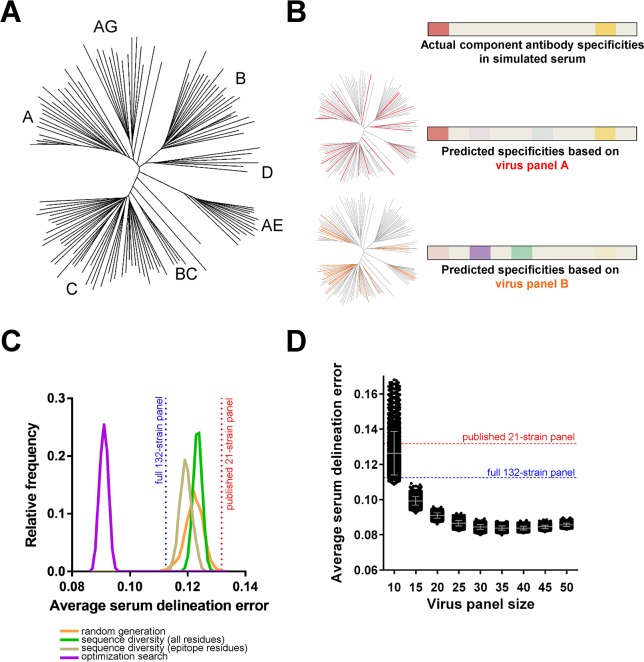
Identification of improved virus panels for NFP analysis. (A) Phylogenetic tree of full set of 132 HIV-1 strains used as a source for virus panel selection. (B) Schematic of virus panel evaluation using simulated sera. Heatmaps show the relative prevalence of actual or predicted antibody specificities in a given simulated serum, highlighting the increased accuracy of NFP predictions when using panel A (red strains) vs. panel B (orange strains). (C) Virus panel search methods using four different approaches (colored curves). For each approach, shown are the relative frequencies (y-axis) of observing virus panels of size 20 with different levels of prediction accuracy (x-axis, average serum delineation error, computed over a set of simulated sera for each virus panel). Highlighted are the serum delineation errors for the published 21-strain panel (red) and the full 132-strain panel (blue). (D) Virus panel size vs. prediction accuracy. Each dot represents the average serum delineation error of a single virus panel, with grey bars representing mean and standard deviation for each virus panel size. Shown are the top 5,000 virus panels for a search method and panel size.

As a starting point for the computational analyses, we compiled neutralization data for a set of monoclonal antibodies against ~200 viruses, and further curated the set of viruses (see Materials and Methods), in order to obtain a starting set of 132 viral strains representing diverse HIV-1 clades (Fig 2A). Candidate virus panels were selected as subsets of the full 132-strain set. For a given virus panel, we used the NFP algorithm to predict the prevalence of different antibody specificities in a set of 4,500 simulated sera. We then compared the predictions to the actual antibody combinations in the simulated sera, in order to obtain the serum delineation error (see Materials and Methods) for the given panel (Fig 2B).

Initially, as a way to evaluate different panel search methods, we focused on identifying virus panels of size 20 (Fig 2C), since that size was deemed to balance between the computational benefit of increased data size and the practical requirement for reduced use of serum samples [4]. As a first step, we generated 100,000 panels by randomly picking sets of 20 strains from the full set of 132 strains. When evaluated on the test set of simulated sera, virtually all of the top 5,000 randomly generated panels had lower average serum delineation error (greater prediction accuracy) compared to the published 21-strain panel (with an average error of 0.13179). Moreover, 27 of these panels had greater prediction accuracy than the full 132-strain panel (with an average serum delineation error of 0.11248), indicating that larger-size panels are not necessarily optimal.

We next explored the hypothesis that optimizing the sequence diversity within a virus panel could result in improved prediction accuracy. To that end, we used an implementation of the A* branch-and-bound algorithm [65] to enumerate virus panels with optimized sequence diversity for either (a) the HIV-1 Env gp140 sequence or (b) only for residues that were part of known antibody epitopes (Materials and Methods). The top 5,000 virus panels from both sequence-derived sets had substantially lower serum delineation errors compared to the published 21-strain panel (Fig 2C). A number of randomly generated panels had lower serum delineation errors than the best virus panels from the full gp140 sequence set, and the medians of the top 5,000 panels for these two distributions were similar (0.12232 for the random set vs. 0.12348 for the gp140 sequence set), suggesting that optimizing the diversity in the full gp140 sequence may not contain sufficient information for improving serum delineation accuracy (Fig 2C). The epitope-derived virus panels appeared to have a more pronounced effect on serum delineation accuracy, with the distribution for the top 5,000 clearly shifted toward lower serum delineation errors, although the best of the epitope-derived panels had error rates comparable to the best randomly generated panels.

Finally, we performed a Monte Carlo-based optimization (see Materials and Methods) to search for 20-strain panels with better performance. Indeed, all of the top 5,000 virus panels from this optimization search were substantially better than the published 21-strain panel, the full 132-strain panel, and the panels from any of the other search methods (Fig 2C).

#### Virus panel size vs. prediction accuracy

While virus panels of size 20 were previously found to represent a good balance between experimental and computational requirements [4], it is possible that panels of other sizes may have more optimal performance. To explore this question, we applied the Monte Carlo-based optimization technique to search for virus panels of size between 10 and 50, at increments of 5, and compared the top 5,000 panels for each panel size (Fig 2D). The panels with size 10 performed notably worse than the other panel sizes, and performance accuracy increased gradually for the panels of size 15–25. Panels of size between 30 and 35 performed the best. Panels of size 40–50 showed slight decreases in performance, although potentially this could be due to undersampling of the larger search space for these panel sizes.

#### Performance advantages of optimized virus panels

**Fig 3:**
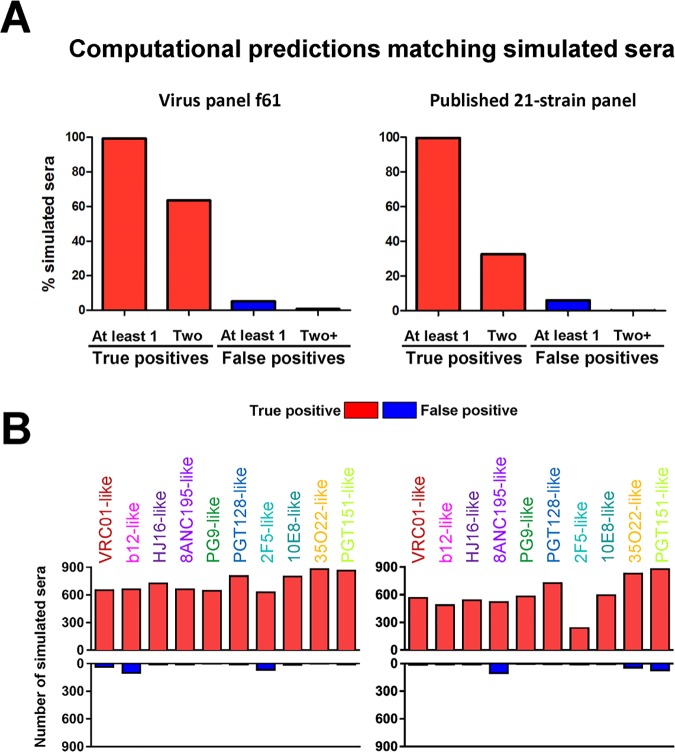
Computational comparison of f61, a selected 20-strain virus panel, to the published 21-strain panel. Both panels were evaluated on the test set of simulated sera. (A) Percent of simulated sera with one or two true positive (red) and one or two+ false positive (blue) signals. (B) Number of simulated sera with true positive (red, out of a maximum of 900) and false positive (blue) signals for each of the ten antibody specificities from the reference set (colored columns).

To gain a better understanding of the advantages of using optimized virus panels, we selected virus panel f61 (see Methods), a promising candidate from the search for 20-strain panels, and compared its performance to that of the published 21-strain panel for the test set of simulated sera (Figs 3 and S3). Panel f61 had high serum delineation accuracy, and contained viruses from diverse clades, in contrast to the published panel which only contained viruses of clades A, B and C. While both panels were able to recover at least one of the two component antibody specificities in virtually all cases (>99% for both panels), f61 successfully identified both component antibody specificities in almost twice as many cases as the 21-strain panel (63.4% vs. 32.5%, respectively) (Fig 3A). The distributions of true and false positives per antibody specificity were also different for the two panels (Figs 3B and S3D). For panel f61, the delineation algorithm successfully identified more than 2/3 of the total number of occurrences (900) for all 10 of the antibody specificities in the 4,500 simulated sera. In contrast, for the published 21-strain panel, 7 of the 10 antibody specificities had less than 2/3 of the possible occurrences successfully identified. For both panels, the identification of 2F5-like specificities had the lowest success rate; for panel f61, however, the rate was similar to that of a number of other specificities, whereas for the 21-strain panel, this rate was substantially lower, at less than 1/3 of the total number of occurrences of 2F5-like specificities (Fig 3B). Of note, the false positive predictions were dominated by different specificities for each of the two panels: b12-like for panel f61 and 8ANC195-like for the 21-strain panel (Fig 3B). However, the number of false positives were on average 27- and 21-fold lower than the number of true positives for the f61 and 21-strain panels, respectively. Taken together, these results confirmed the utility of both panels for use in NFP-based serum delineation, with notable advantages of using the new f61 virus panel.

**Fig 4:**
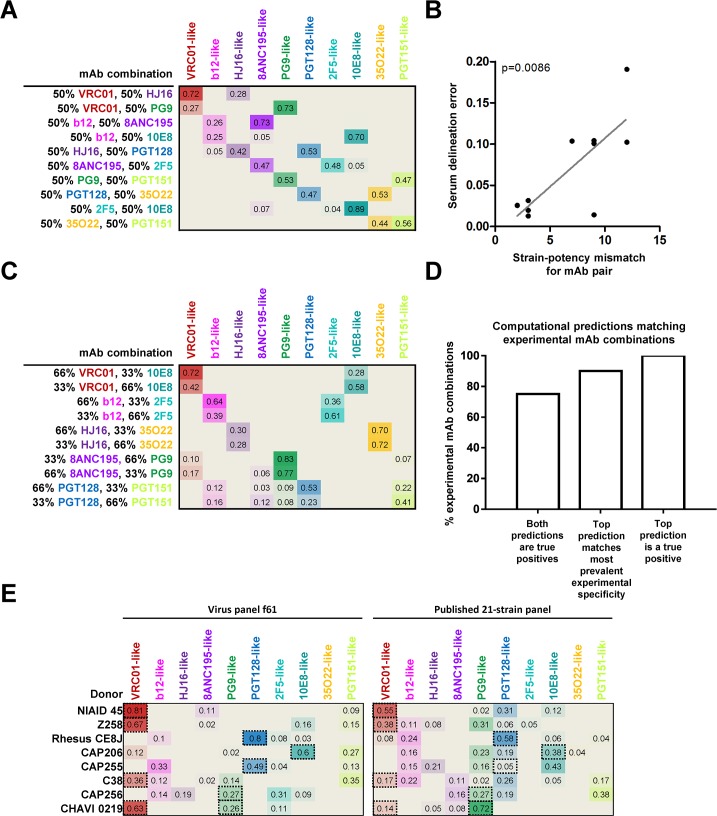
NFP analysis of mAb combinations and donor sera for virus panel f61. (A) NFP predictions for ten pairwise mAb combinations (rows), 1:1 ratio. Values shown are the NFP delineation scores for each of ten antibody specificities (columns) in each mAb combination; colored heatmap is proportional to the respective delineation scores. (B) Correlation between serum delineation error and strain-potency mismatch for the ten mAb pairs from (A). (C) NFP predictions for ten pairwise mAb combinations, 2:1 ratio. (D) Agreement between NFP predictions and actual specificities for the mAb combinations in (A,C). (E) NFP analysis of eight donor sera against f61 and the published 21-strain panel. Dotted-line boxes highlight experimentally confirmed specificities.

To further evaluate panel f61, we generated experimental neutralization data for that panel against a set of monoclonal antibody combinations and polyclonal donor sera (Figs 4 and S6). In the first set of experiments, pairs of antibodies were mixed in a 1:1 ratio to obtain pairwise combinations, with one antibody chosen from each of 10 reference specificities. NFP was able to predict a true positive signal for all combinations, and both specificities were successfully identified in all but one of the combinations (Fig 4A). We further observed that the accuracy of the NFP predictions was directly correlated with how dominant one of the antibodies was in a given pair–as might be expected, accuracy decreased for mismatched pairs where one antibody was substantially more potent (and thus dominated the polyclonal neutralization signal) (Fig 4B). We also assessed the ability of NFP to identify signals in combinations where antibodies were mixed in unequal ratios. To that end, we obtained neutralization data for pairwise combinations in a 2:1 ratio. Again, NFP identified a true positive signal for all combinations, and the top NFP prediction corresponded to the more prevalent specificity for eight of the ten combinations (Fig 4C). Overall, NFP identified at least one of the true specificities for all twenty tested pairwise combinations, and both specificities for 75% of the tested combinations (Fig 4D). Taken together, the analysis of the monoclonal antibody combination data suggested that NFP is capable of identifying antibody specificities that are substantial contributors to the neutralization signals of polyclonal sera.

Next, we evaluated panel f61 on data for eight serum samples with previously characterized specificities (including bNAbs isolated from seven of the donors), and compared the resulting NFP predictions to those from the published 21-strain panel (Fig 4E). For three of the sera (NIAID 45, Z258, and rhesus CE8J), panel f61 yielded an exclusive signal for a specificity that is known to be dominant in the given sample [9, 66–68]; the published 21-strain panel identified the respective specificities as well, but secondary signals were also observed, despite a lack of experimental evidence suggesting multiple broadly neutralizing specificities. For donor CAP206, the f61 panel identified a substantially stronger signal (compared to the published 21-strain panel) for the experimentally confirmed 10E8-like specificity [69], although a secondary signal was also observed. In the case of CAP255 and C38, only the f61 panel successfully identified signals for experimentally confirmed specificities (VRC01-like for C38 [68] and PGT128-like for CAP255 [37]), whereas the 21-strain panel could not. For donor CAP256, the expected PG9-like signals [14, 46] were successfully identified by both panels, although in both cases signals for additional unconfirmed specificities were also observed. For donor CHAVI 0219, from which both PG9-like and VRC01-like antibodies have been isolated [18], the published 21-strain panel only identified a PG9-like signal, while panel f61 was able to identify both. This finding is in agreement with the advantages that we observed in the computational assessment of panel f61 (Fig 3A), and specifically, with the improved ability of this panel to recognize multiple neutralization signals in polyclonal sera.

### Identification of sera with potentially novel bNAb specificities

**Fig 5:**
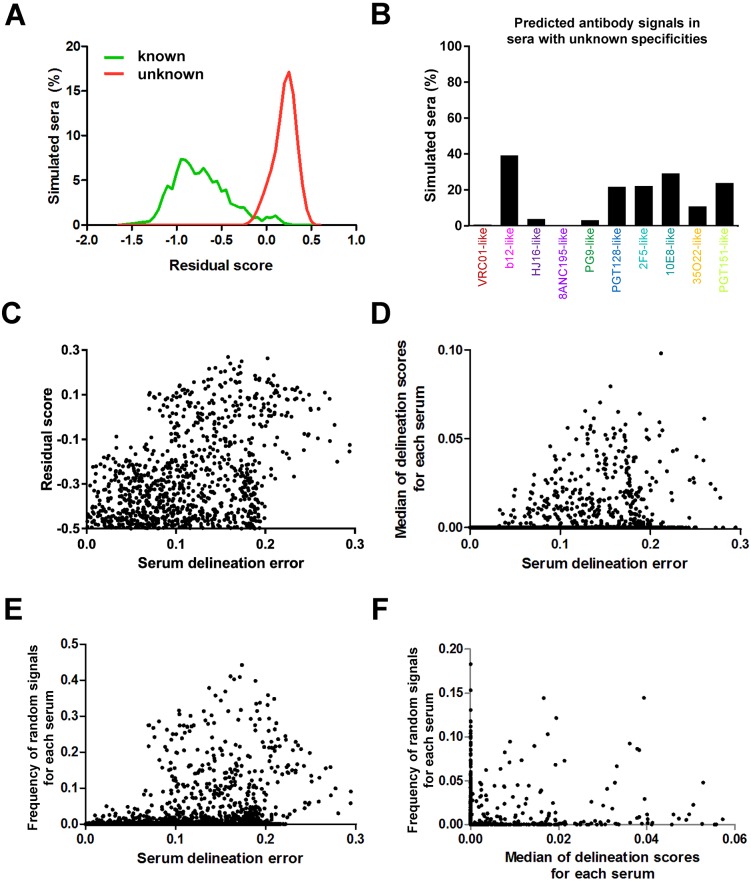
Prediction of potentially novel specificities and confidence scores in NFP analysis with virus panel f61. (A) Histograms of residual scores for simulated sera with dominant known (green) vs. unknown (red) specificities. (B) Frequency of predicted known antibody signals (colored) for simulated sera with unknown specificities. (C-E) Relationship between serum delineation error (a measure of NFP prediction accuracy) and three confidence scores: (C) residual scores (for values of at least -0.5), (D) median of the ten delineation scores, and (E) frequency of random signals for a set of simulated sera with dominant known antibody specificities (dots). For each of the three confidence scores, higher values were generally associated with greater serum delineation errors. (F) Complementarity of confidence scores. Shown are the frequency of random signals and median of delineation scores for simulated sera (dots) with residual scores less than -0.1.

While many polyclonal responses against HIV-1 can be attributed to known antibody specificities, new broadly neutralizing epitopes continue to be discovered [11, 12, 19] and there are samples whose neutralizing activity cannot be explained by known specificities [35, 70]. The first-generation of NFP algorithms, however, are only capable of predicting specificities that are already part of the reference set of known antibodies (S1A Fig). To address this challenge, we sought to develop algorithms for predicting the existence of dominant potentially novel bNAb specificities in a given sample. To that end, we used virus panel f61 to generate simulated sera with neutralization patterns that had virtually no correlation with any of the neutralization fingerprints from the reference bNAb set (see Materials and Methods), and applied the NFP algorithm to deconvolute these sera. We used normalized residual scores as a measure of the fit between the neutralization fingerprints of the reference bNAbs and the neutralization pattern of a given serum (see Materials and Methods). The residual scores for the sera with unknown specificities were significantly different from the scores for the test set of sera with known specificities (p<0.0001, Mann-Whitney test), with only minor overlap between the two distributions (Fig 5A). In fact, ~95.5% of the sera with known specificities had residual scores of at most -0.1 (median: -0.7704; range: -1.617 to 0.2605), whereas more than 98% of the sera with unknown specificities had residual scores greater than -0.1 (median: 0.2172; range: -0.2719 to 0.5527). These results confirmed that high NFP residual scores can indicate the existence of potentially novel specificities in a given serum.

Analysis of the predicted signals for the simulated sera with unknown specificities revealed that b12-like signals were by far most common (~40%) (Fig 5B). This was in agreement with the observation that the highest false positive rate for the sera with known specificities was for b12-like signals (Fig 3B). To determine whether some specificities are more likely to be predicted as particular other specificities, we analyzed the correspondence between false negatives and false positives in the set of simulated sera (S4 Fig). The results indicated that, for most of the other specificities, b12-like signals were the most common false positives for sera with false negatives. To determine whether the assignment to b12-like signals may preferentially occur for limited-breadth sera that may share a large number of neutralization-resistant values with the b12 fingerprints, we analyzed the variation in observed signals for increasing serum neutralization breadth for the sera with unknown specificities (S5 Fig). The prediction frequency of b12-like signals was generally similar for all levels of serum neutralization breadth, and similar consistencies were observed for the other nine specificities, with b12-like signals being the most common for all levels of serum neutralization breadth (S5 Fig). Taken together, at least in the case of virus panel f61, these results may indicate that the b12-like neutralization fingerprint can play a role as a sink for neutralization signals that may not be b12-related but that are difficult for the NFP algorithm to deconvolute.

### Confidence scores for the computational predictions

Even if a given polyclonal serum is dominated only by already known antibody specificities, the NFP algorithm may in some cases still result in inaccurate predictions, in the form of, e.g, false positives or false negatives (Fig 3). It would therefore be helpful to develop additional algorithms that can estimate the confidence in the NFP predictions for a given serum. To that end, we focused our analysis on the set of simulated sera with known specificities, in order to identify potential markers of NFP prediction confidence. The residual scores discussed above appeared to be a good indicator of prediction confidence, with high residual scores typically associated with high serum delineation error (Fig 5C). In addition, the median of the delineation scores for a given serum was also found to be associated with the magnitude of the serum delineation error (Fig 5D). Finally, we observed that the frequency with which random neutralization fingerprint signals were observed for a given serum was also generally associated with how accurate the computational predictions are (Fig 5E). The correlation between serum delineation error with each of the three measures was significant (p<0.0001, Spearman). Moreover, each of the three measures was able to contribute to the identification of less reliable predictions not identified by the other two measures (Fig 5F).

To interrogate the usefulness of these scores for real data, we analyzed the results from panel f61 for donor and rhesus macaque sera (Fig 4E), and observed that only the predictions for donors CAP256, C38, and CAP255 were associated with lower confidence (S7 Fig), in agreement with the existence of a relatively strong unconfirmed signal in these predictions (Fig 4E). The scores for the other five donors were associated with high confidence (S7 Fig), reflecting the agreement with the dominant signals for experimentally confirmed specificities for these donors (Fig 4E). Taken together, these results suggest that residual scores, median of delineation scores, and frequency of random signals can all be useful as criteria for determining the confidence in the NFP predictions.

### Algorithm for cohort-level NFP analysis

Knowledge about the common specificities found in HIV-1 infected individuals and participants in vaccine trials can provide valuable insights into the interactions between virus and host at the cohort, or even population, level. While it is possible to use experimental mapping to delineate the antibody specificities observed in large cohorts [70], the efficiency of the neutralization-based NFP approach makes it particularly well-suited to such large-scale, cohort-level, analysis. To assess the applicability of NFP to cohort-level analysis, we developed a simulation procedure in which multiple cohorts of simulated sera were generated, and the ability of NFP to predict the overall prevalence of the different antibody specificities within each cohort was evaluated (see Materials and Methods).

**Fig 6:**
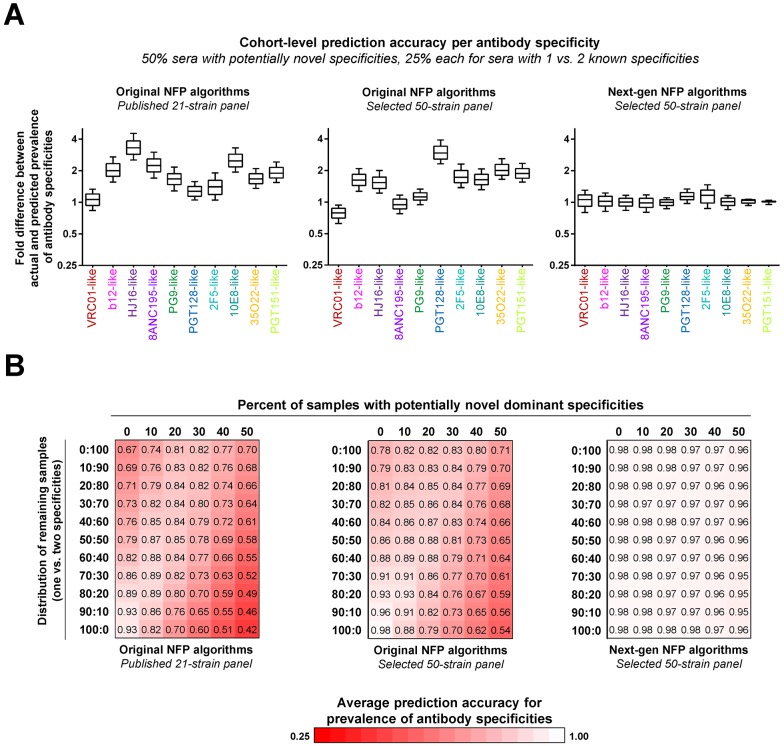
Simulation of cohort-level analysis of polyclonal antibody responses. Three sets of NFP analyses were performed: (left) original NFP algorithms with the published 21-strain panel, (middle) original NFP algorithms with a selected 50-strain panel, and (right) next-generation NFP algorithms with the selected 50-strain panel. (A) Analysis for a simulated sample with 50% sera with potentially novel specificities, and 25% each of sera with 1 or 2 dominant known specificities. For each antibody specificity from the reference set (colored labels), shown are fold differences between actual and predicted prevalence in 1,000 samples of 200 sera each. Boxes represent 25–75 percentiles of the data, with vertical bars corresponding to the 10–90 percentiles, and the median shown as a horizontal line within each box. (B) Analysis for different distributions of simulated sera with known (rows) vs. potentially novel (columns) specificities. Each value shown is the average of the median fold differences for each of the ten specificities given the respective distribution of sera. Values are colored as a red-white heatmap.

Initially, we focused on simulated cohorts in which half of the simulated sera had dominant unknown antibody specificities, while the remaining sera in the cohort was divided in 1:1 ratio of sera with one vs. two dominant known specificities (Fig 6A). Since f61 was not a subset of previously published large-panel serum-virus neutralization data [63], we applied our virus panel search algorithm and identified an optimized 50-strain panel for use in the cohort-level analysis (see Materials and Methods). We observed that using the original, first-generation, NFP algorithms with the optimized virus panel resulted in slightly better overall accuracy compared to the published 21-strain panel (Fig 6A, left and middle). To improve the results further, we applied the next-generation NFP algorithms, which use the same calculation of delineation scores as the first-generation NFP algorithms, and then incorporate filters based on residual scores, median of delineation scores, and frequency of random signals as described above. In addition, we applied a procedure specifically designed to incorporate expected algorithm accuracy into the cohort-level analysis (see Materials and Methods). This next-generation NFP analysis on average resulted in excellent agreement between predicted and actual prevalence of the different antibody specificities within the samples of simulated sera (Fig 6A, right). Notably, while the selection of an optimized virus panel resulted only in a slight improvement of the accuracy of the cohort-level analysis (p = 0.2754, Wilcoxon), the use of the next-generation (as opposed to the first-generation) NFP algorithms led to a dramatic improvement in the results (p = 0.002, Wilcoxon).

Since the actual relative occurrence of donor sera with unknown specificities vs. sera with known specificities is unclear and may vary depending on the specific cohort (e.g., duration of infection, dominant HIV-1 subtypes, etc.), we next assessed different distributions of the simulated serum samples, ranging from 0% to 50% occurrence of sera with unknown specificities, with the remaining sera divided between sera with one vs. two dominant specificities (Fig 6B). The first-generation NFP algorithms with the optimized virus panel outperformed the published 21-strain panel for all of the different sample distributions (Fig 6B, left and middle). In both cases, the worst performance was observed for samples with a high percentage of sera with unknown specificities combined with sera with a single dominant known specificity. As expected, the best performance was associated with samples consisting only of sera with one dominant known specificity (and no sera with unknown or two known dominant specificities) (Fig 6B, left and middle). In contrast, the next-generation NFP algorithms on average showed excellent accuracy for all of the different sample distributions (Fig 6B, right). Taken together, these results suggested that the next-generation NFP algorithms are appropriate for cohort-level analysis of antibody responses against HIV-1.

### Large-scale analysis of polyclonal antibody responses against HIV-1

**Fig 7:**
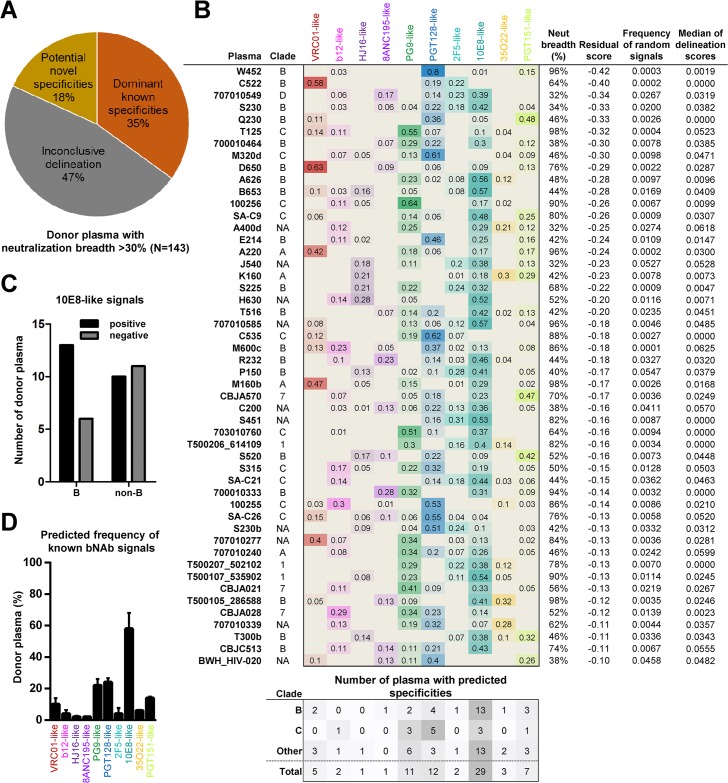
Large-scale analysis of HIV-infected donors. (A) NFP predictions for 143 donor plasma were divided into three groups: plasma with dominant known specificities, with potentially novel specificities, or with inconclusive delineation. (B) Delineated antibody specificities for the 50 plasma with predicted dominant known specificities. Also shown are the clade of infection (A, B, C, D; 1: CRF 01; 7: CRF 07; NA: unknown), neutralization breadth, as well as the three confidence scores, for each sample. The number of positive signals for clades B and C are shown at the bottom, with the ‘Other’ category including both non-B and non-C as well as samples with unknown clades. (C) Prevalence of 10E8-like signals in clade B vs. non-B samples. (D) Overall prevalence of antibody specificities (thick bars) in the 50 samples from (B), and adjusted predictions after the application of the cohort-level NFP algorithm (thin bars). The analysis was based on the selected 50-strain panel from Fig 6.

We next sought to apply the next-generation NFP algorithms to the analysis of plasma samples from a large collection of HIV-infected donors from diverse clades of infection. To that end, we compiled published neutralization data for ~200 donors [63], which was then reduced to a final set of 143 donors with neutralization breadth of at least 30% (Figs 7 and S8). Approximately one fifth of the sera were predicted to have dominant potentially novel specificities (associated with high residual scores), while approximately one third of the sera were predicted to have one or more dominant known specificities based on good confidence scores (Fig 7A and 7B, and Materials and Methods). The predictions for the remaining approximately one half of all sera were deemed inconclusive, since at least one of the respective confidence scores was not within the defined threshold (Figs 7A and S8C, and Materials and Methods). For the sera with predicted known specificities, all of the different types of known antibody specificities were observed, albeit with different frequencies (Fig 7B). Moreover, the specificity predictions appeared to be associated, to an extent, with the clade of the donor’s infection (Fig 7B). In the case of clade C plasmas, PGT128-like specificities were the most common, followed by PG9-like and 10E8-like specificities, although the small number of clade-C samples may be a limiting factor in this analysis. In contrast, 10E8-like signals were by far the most common in clade-B samples (Fig 7B). In fact, although 10E8-like specificities were predicted in both clade-B and non-clade-B samples, an enrichment of 10E8-like signals was observed in clade-B samples (Fig 7C). Overall, for the collection of samples analyzed here, 10E8-like specificities were the most common, followed by PGT128-like and PG9-like specificities (Fig 7D). Taken together, these results show the potential of using the NFP approach for large-scale analysis of antibody responses against HIV-1.

## Discussion

Computational analysis of virus neutralization data is now an established method for mapping the antibody specificities found in polyclonal responses against HIV-1. The success of NFP and other computational approaches for analyzing the interactions between HIV-1 and the immune system [4, 41, 58, 71] underlines the power and potential of computation to develop transformative technologies with significant biological impact. One of the major challenges for such approaches, however, has been the limited amounts of available data. In the case of NFP, while matrices of several hundred virus strains against several hundred monoclonal or polyclonal antibodies are pushing the boundaries of what is feasible experimentally, advanced computational techniques such as machine learning would benefit immensely from the existence of substantially larger training datasets. To address this issue, we developed a framework for simulating polyclonal antibody responses. While antibody responses to HIV-1 are complex and polyclonal [72], there is limited to no evidence to suggest that within any given serum there are a large number of different antibody specificities that each achieve broad neutralization [12, 14, 18, 66]. We therefore elected to utilize simulations of pairwise monoclonal antibody combinations in the current study, although the simulation framework here can be extended to accommodate higher-order polyclonal simulations. Advances in the modeling of neutralization data (e.g., [73] or the addition of modeling for antibody synergism or antagonism) can lead to further improvements in the simulation framework, and consequently, the accuracy of the predictive algorithms. Overall, the ability to build general computational platforms that simulate or mimic experimental assays of interest (such as antibody-virus neutralization data) can provide an efficient means to generate otherwise infeasible large-scale datasets, opening the door for new technology developments and novel biological insights.

Here, we developed next-generation technology that addresses a number of key challenges, significantly advancing the utility of the neutralization-based NFP approach and enabling the investigation of important new biological questions, for analysis both at the individual donor level and for entire cohorts. These two types of analyses, however, have notably different goals. At the individual donor level, the primary goal is the successful identification of antibody neutralization signals in a given polyclonal response, typically with the goal of performing subsequent antibody isolation and characterization. Improving the accuracy and confidence in the computational predictions is thus essential for this type of analysis. To that end, we developed methods for the identification of sera with potentially novel specificities, as well as for assessing the confidence in the computational predictions for a given sample. In addition, we developed an approach for selecting viral strains for neutralization experiments that improve the ability of the algorithm to predict component antibody specificities in polyclonal responses. Virus panel size and selection was found to be significant for the accuracy of the NFP predictions—for example, the delineation for donor CAP256 (also called 100256), was much cleaner (a single positive PG9-like signal) when using a 50-strain panel vs. either the 20-strain f61 or the published 21-strain panel (Figs 4E and 7B). However, while our results indicated that increasing the size of the virus panels, at least up to a certain level, may contribute significantly to the accuracy of the computational predictions, practical considerations such as limited sample availability or feasibility of large-scale neutralization experiments may in many cases still necessitate the use of virus panels of size 20 or less, which can still exhibit excellent levels of prediction accuracy. In addition, the large 132-strain panel was found to be less optimal for NFP analysis compared to many smaller-size panels identified in our search. This observation could potentially be due to favoring broader vs. weaker antibodies when using large panels, or to an increased unnecessary redundancy in large panels. Since the NFP method is affected, in part, by the ability to discriminate between the neutralization fingerprints of antibodies with different epitopes vs. antibodies with similar epitopes, it is not surprising that ad hoc selection of virus strains may not lead to optimal prediction accuracy. Overall, the results from the virus panel analysis performed here emphasize the importance of virus panel selection for neutralization analysis. Generally, the virus panel selection method here can be adapted to the particular problem at hand, such as selecting a subset of strains from already existing neutralization data, or performing clade-specific strain selection. In addition, as novel anti-HIV antibodies continue to be discovered and thereby improve our understanding of the major neutralizing epitopes, this method provides a framework for assessing virus panels to be used with alternate sets of antibodies as templates for serum specificities.

At the cohort or population level, the primary goal is to accurately predict the overall prevalence of different types of antibody specificities in the analyzed samples. To achieve this, in addition to the technology developed for improving individual-level analysis, we introduced new algorithms specifically designed for handling collections of samples. A potential additional application of population-level analyses would be for quantifying the advantages of using the NFP method for serum delineation. For example, by using the predicted frequency of occurrence of the different bNAb specificities (Fig 7D), the probability of choosing the correct signals for individual donor sera can be computed: e.g., 0.016 for identifying a single positive (VRC01-like) signal for donor 45 and 0.006 for identifying two signals (VRC01-like and PG9-like) for CHAVI 0219. Such quantification analyses can further underline the utility of using the NFP approach for delineating polyclonal antibody responses to HIV-1 infection.

The large-scale HIV-infected cohort analysis provides initial evidence that NFP can be adapted to cohort-level analysis, although some interesting trends, such as potentially clade-specific variation of the predicted antibody responses, were also observed with the data in the current study. In particular, the high frequency of 10E8-like signals was an interesting observation (Fig 7B and 7D). While NFP analysis of additional HIV infection cohorts and further experimental mapping will help clarify the overall prevalence of 10E8-like antibodies, the fact that false positive 10E8-like signals were not common in our simulations of sera consisting of known bNAbs (S8B Fig) and the various filters applied to remove sera with potentially inaccurate predictions, act in support of the bNAb prevalence observations for the samples shown in Fig 7B. Expanding the population-level analysis to a larger set of samples, with increased diversity that more closely reflects the actual global distribution of infections, will be informative for deciphering potential relationships between the phenotype of the neutralizing antibody response and the genetic characteristics of the infecting virus. Ultimately, such analyses can help guide the design of epitope-specific HIV-1 vaccines that are tailored to take into account the prevalence of infecting clades within a specific geographic region.

## Materials and Methods

### Neutralization data

Single round of replication Env-pseudoviruses were prepared, titered and used to infect TZM-bl target cells (obtained from George Shaw, University of Pennsylvania) as described previously [74]. Neutralization data of monoclonals was determined using a panel of ~200 geographically and genetically diverse Env-pseudoviruses representing the major subtypes and circulating recombinant forms. The data were calculated as a reduction in luminescence units compared with control wells, and reported as half-maximum inhibitory concentration (IC_50_) in micrograms per milliliter for monoclonal antibodies, or reciprocal dilution (ID_50_) for serum samples. Since the antibody-virus neutralization matrix had missing data (i.e., no available data for a subset of the antibody-virus pairs), the initial set of ~200 HIV-1 strains was filtered to the largest common subset of strains with data for antibodies from all known major broadly neutralizing epitopes. That subset was further filtered to exclude strains for which data showed they were easy to neutralize by polyclonal samples or by weakly neutralizing monoclonal antibodies, as well as strains that had been noted to have yielded inconsistent data in previous experiments with serum. This filtering process resulted in a panel of 132 strains that were used in the analysis. The reference set of bNAb specificities was constructed to include antibodies from the known major sites of vulnerability. Specifically, antibodies were grouped into ten epitope-specific clusters, with each cluster represented by one or more antibodies. An antibody neutralization breadth of at least 30% was used as a cutoff for inclusion in the reference antibody dataset. Further, only one antibody from a given lineage was included in the reference set, and multiple repeats of the same antibody-virus neutralization data were averaged. The final reference set consisted of 22 antibodies with well-characterized epitopes, as follows: CD4 binding site—VRC01-like: VRC01, VRC27, VRC-CH31, VRC-PG20, VRC-PG04, VRC23, 12A12, 3BNC117; b12-like: b12; HJ16-like: HJ16. V1V2 apex—PG9-like: PG9, PGT145, CH01. Glycan-V3—PGT128-like: PGT121, PGT128, PGT135. Membrane-proximal external region - 2F5-like: 2F5; 10E8-like:10E8, 4E10. The gp120-gp41 interface - 35O22-like: 35O22; 8ANC195-like: 8ANC195; PGT151-like: PGT151. These ten antibody groups differed compared to [4], reflecting the discovery of new broadly neutralizing antibody epitope specificities since the original NFP publication. For each antibody, the neutralization fingerprint was defined as the IC_50_ values for that antibody against the set of HIV-1 strains. Neutralization data against selected viral strains was generated for the following serum samples: NIAID 45 and Z258 (from Mark Connors, NIAID), C38 (from VRC, NIAID), Rhesus CE8J (from Malcolm Martin, NIAID), CAP256, CAP255, and CAP206 (from Lynn Morris, CAPRISA), and CHAVI 0219 (from Barton Haynes, Duke-CHAVI-ID).

### NFP analysis of polyclonal antibody neutralization

The prediction of antibody specificities from polyclonal neutralization was performed as described previously [4] (S1A Fig). Briefly, polyclonal neutralization on a set of HIV-1 strains was represented as a combination of the neutralization fingerprints of the ten epitope-specific clusters from the antibody reference set. The ten epitope-specific clusters were formed by performing antibody clustering based on the neutralization fingerprints, as described previously [4]. A least-squares fitting procedure was then used to estimate the relative contribution to polyclonal neutralization of each of the ten epitope-specific antibody specificities. Effectively, for a given serum, this procedure results in a list of ten coefficients (one for each antibody specificity), each ranging from 0 to 1, that sum to 1; output coefficients (referred to as delineation scores for each antibody specificity) of more than 0.25 were considered as positive signals, therefore allowing at most four positive signals in any given serum. A new Octave implementation of the algorithm was used, and additional functionality and scripts were implemented in Mathematica and Java. The next-generation NFP technology uses the same polyclonal specificity prediction module as the original NFP [4]. However, the next-generation NFP incorporates a number of new algorithms compared to the original NFP: algorithms for predicting the presence of potentially novel specificities; algorithms for reporting prediction confidence scores; algorithms for population-level analysis; and algorithms for selection of improved virus panels for neutralization experiments.

### Generation of simulated sera

Simulated sera were generated as pairwise bNAb combinations that mixed equal amounts of an antibody representative from two of the ten epitope-specific antibody groups. Two sets of 4,500 simulated sera were generated: (i) one set of sera was used as a training set for the virus panel search procedure, while (ii) the other set was used as a test set for evaluating the performance of candidate virus panels identified during the search. The training set included pairwise combinations between antibodies from the reference set. The test set included an expanded set of 56 antibodies that consisted of clonal variants of antibodies from the reference set, as well as additional antibodies targeting epitopes that generally overlapped with, and showed similar neutralization fingerprints to, one of the epitope-specific clusters from the reference set (S9 Fig). Of note, the neutralization-based antibody clustering was not found to be dependent on the antibody neutralization breadth: antibodies with substantially different neutralization breadth were successfully clustered close to the appropriate groups (for example, CAP256-VRC26.03 with 38% and PG9 with 79%); similar observations were made for antibody development intermediates with limited breadth [75]. Due to the limited number of bNAbs for which there exist large-scale antibody-virus neutralization data, especially for classes where only one bNAb representative has been characterized (such as 35O22), it is not possible to avoid the inclusion of antibodies in the test set of sera that are similar to or the same as the antibodies in the training set. However, because of the fingerprint transformation methods for serum simulations (described below), the training and test sets of sera would be diverse even if using the same set of bNAbs. In addition, the expansion of the antibody set for the test sera allows for further improvement in serum diversity. Analysis of the serum delineation error for the top 5,000 20-strain panels against test sera that either include bNAb clonal relatives (the 56-antibody set described above) or include only a single representative from each clonal group showed a significant correlation (p<0.0001, Spearman; S10 Fig). Overall, while the test set of sera are not optimal due to the use of antibodies that overlap the training set, these test sera provide a useful quantitative measure for comparison of virus panel performance.

For each pairwise combination, an antibody was selected from two of the ten epitope-specific clusters, and the neutralization fingerprint for the resulting combination was obtained as follows. First, the neutralization fingerprint for each of the two antibodies was transformed using a simulation of variability for the neutralization experiments. Specifically, a set of actual experimental variability values observed for multiple repeats of the same neutralization experiments (for example, for multiple repeats of the neutralization of strain YU2 by antibody VRC01) was compiled; then for each *n(a*,*v)* (the measured neutralization IC_50_ value for antibody *a* against viral strain *v*), a scaling factor *k*_*1*_ was obtained from a distribution formed by the set of experimental variability values (using the *EmpiricalDistribution* function of Mathematica). Next, the potency of the resulting adjusted fingerprints for each of the two antibodies were scaled by a random factor *k*_*2*_ between 1/10 and 10, where *k*_*2*_ was constant for all *n(a*,*v)* for antibody *a* in the selected antibody pair. These two transformations (to account for experimental variability and to incorporate a notion of potency variation between antibodies with similar epitopes) aim at increasing the diversity of the simulated fingerprints. Finally, the two transformed fingerprints were combined by taking the minimum of the two neutralization values for each of the HIV-1 strains, in order to obtain the neutralization fingerprint for the simulated serum. Specifically, given two selected antibodies *a*_*1*_ and *a*_*2*_ and a specific viral strain *v*, the neutralization value for the simulated serum for the combination of *a*_*1*_ and *a*_*2*_ is computed as: *min [t(n(a*_*1*_,*v))*, *t(n(a*_*2*_,*v))]*, where *t(x) = k*_*1*_ *k*_*2*_ *x* is the transformation of the original antibody-virus neutralization data *x*, as described above.

Taking the minimum of the two neutralization values for an antibody pair within a combination is a modeling simplification of monoclonal antibody interactions in polyclonal sera. In reality, antibodies in serum would have complex interactions that could be antagonistic (such as for antibodies that bind to different, incompatible, Env conformations), synergistic, or in some cases–independent. Ideally, a serum simulation model would be able to accurately account for such interactions, but current knowledge about the determinants of these interactions is limited, and such model improvements will be the aim of future work on simulating polyclonal serum neutralization. Nevertheless, despite the modeling simplifications, the excellent agreement between simulated and actual neutralization fingerprints (Figs 1B and S2B) suggests that the current model is appropriate for modeling serum simulations. Interestingly, for two antibody combinations in which the two antibodies in each combination had similar or overlapping epitopes (VRC01+HJ16 and 2F5+10E8, Fig 4A), the experimentally determined and the computationally predicted neutralization values were in excellent agreement (Spearman correlations of 0.96 and 0.98, respectively), further underlining the feasibility of the proposed serum simulation procedure. The generated simulated sera represented diverse levels of neutralization breadth (S2C and S3B Figs).

Sera with potentially novel (or unknown) specificities were computed by identifying random neutralization fingerprints that, for a given virus panel, had low (<0.2) Spearman correlation with any of the antibody neutralization fingerprints from the reference set; a total of 10,000 such simulated sera were used in the analysis. While it is possible to specifically optimize virus panels for detection of novel specificities, we used virus panel f61 for this analysis because of its improved performance with the known bNAb specificities, which would likely be commonly found in serum analysis.

### Computation of serum delineation error

For a given virus panel, reference antibody set, and simulated sera, the average serum delineation error was computed as follows. For each simulated serum, the NFP algorithm was applied to generate predictions about the neutralization prevalence of each of the antibody specificities from the reference set: we refer to these predictions as the NFP delineation scores. The serum delineation error for a given simulated serum was then computed as the RMSD between the NFP delineation scores and the actual antibody composition in that serum. The average serum delineation error was computed as the average of the RMSD’s for the entire set of sera. The serum delineation error was used as a measure to determine the prediction accuracy of a given virus panel.

### Virus panel search

Several methods were used for searching for optimized virus panels of size 20. *Random search*. Virus panels were generated by randomly selecting subsets of size 20 from the 132 strains. *Sequence diversity*. HIV-1 Env sequence analysis for the 132 strains was performed either for all residues between 1–683 or only for a subset of the residues that were found to be part of antibody epitopes from antibody-antigen complex structures. In each case, the selected Env sub-sequences were grouped into 20 clusters based on sequence similarity [4]. The A* search algorithm [65] was then applied by constraining enumeration of candidate panels to include a single strain from each of the 20 clusters. Given these constraints, the A* algorithm generated a set of virus panels with optimized sequence diversity. *Monte Carlo-based optimization search*. Multiple starting points were each initialized to a random selection of 20 strains from the full set of 132 strains, and an optimization search was performed for each starting point. For each starting point, each step of the search procedure consisted of randomly replacing one of the strains in the current 20-strain panel, and retaining the new panel if a success criterion was achieved, or otherwise discarding the replacement and keeping the previous panel. The success criterion was determined by comparing the prediction accuracy (serum delineation error) over the set of training sera for the new vs. previous panel: the new panel was retained either if its prediction accuracy was better, or with a probability dependent on the difference in prediction accuracy between the two panels. For each search method, a selected set of top virus panels was evaluated on the independent set of test sera that was not used during the search procedure.

When selecting a specific virus panel for neutralization experiments, additional criteria could be utilized, such as ability to use neutralization fingerprints to successfully cluster antibodies consistent with epitope similarity (S3A Fig), clade distribution of selected strains, rate of false positive predictions, minimum antibody neutralization breadth over the given panel, etc. In particular, an antibody clustering procedure analogous to the procedure described in [4] can be used for filtering out candidate virus panels that do not correctly cluster the neutralization fingerprints of antibodies according to epitope similarity. Specifically, for a given virus panel, neutralization-based antibody clustering is performed using the *Agglomerate* function in Mathematica with default parameters (squared Euclidean distance and single linkage). The resulting hierarchical clustering is then compared to the ten pre-defined epitope-specific antibody groups by using the *ClusterSplit* function in Mathematica, and virus panels that misclassify antibodies (e.g., S3A Fig) are discarded from further consideration.

In the case of the 20-strain virus panel f61, that panel was selected based on a combination of criteria, including a good (though not the best) serum delineation accuracy among the panels identified in the 20-strain panel optimization search, ability to correctly cluster the neutralization fingerprints of reference bNAbs according to epitope specificity (S3A Fig), high minimum bNAb neutralization breadth (to allow for a non-negligible serum neutralization signal from each respective antibody group), and sequence diversity (for example, presence of at least two strains each of clades A, B, and C), among others.

### Computation of strain-potency mismatch for mAb pair

For a given pair of monoclonal antibodies *a*_*1*_ and *a*_*2*_, the strain-potency mismatch value was defined as the absolute value of the difference between the number of strains for which *a*_*1*_ is more potent and the number of strains for which *a*_*2*_ is more potent. Pearson correlation and p-value were computed for strain-potency dominance vs. NFP serum delineation error.

### Computation of residual scores

For a given simulated serum, the residuals from the fit of the serum neutralization fingerprint and the combination of reference-antibody fingerprints resulting from the NFP delineation were computed. For a given virus panel, the residual score for a given serum was obtained as a sum of two t-scores based on the computed residuals, divided by a factor of 1000: (a) a t-score computed over the set of sera with known specificities, and (b) a t-score computed over the set of sera with potentially novel (or unknown) specificities.

### Computation of confidence scores

In addition to residual scores, two other scores were defined as means to estimate the confidence in the computational predictions for a given serum. *Median of delineation scores*: computed as the median of the NFP delineation scores for the ten specificities from the reference antibody set. *Frequency of random signals*: For a given serum, the frequency of random neutralization signals was computed as follows. The reference set of ten epitope-specific antibody specificities was expanded to include an eleventh specificity. For each serum, NFP delineation was performed by incorporating, separately, each of the (10,000) random neutralization fingerprints for simulated sera with unknown specificities as the eleventh reference specificity. Then, for each serum, the frequency of random signals was computed as the fraction of cases in which the eleventh (random) specificity had a positive signal, within the 10,000 NFP runs for the given serum.

### Algorithm for cohort-level NFP analysis

Sets of sera, either with unknown antibody specificities or with 1 or 2 dominant known antibody specificities (with the assumption that sera with more than 2 dominant broadly neutralizing specificities would not be common [12, 14, 18, 66]), were generated using the algorithms described above. From these, multiple simulations, with different fractions of sera with unknown specificities vs. sera with 1 or 2 known antibody specificities, were performed. In each simulation, a sample size of 200 sera was used. Sera with unknown specificities were allowed to represent 0 to 50%, at increments of 10%, of the 200 sera, while the remaining set of sera was distributed between sera with 1 vs. 2 specificities at a ratio ranging from 0:100% to 100:0%, at steps of 10%. For a fixed distribution of serum specificities, 1,000 samples of 200 sera each were generated. NFP analysis was performed for each of the 1,000 samples, and the prediction accuracy for the prevalence of each of the ten antibody specificities was computed as follows. For each of the 1,000 samples, the ratio of the NFP-predicted prevalence of a given antibody specificity and the actual prevalence in the respective set of simulated sera (after filtering of sera based on confidence scores, if using the next-generation NFP) was reported as the fold difference between actual and predicted prevalence for a given antibody specificity. The fold differences were presented either as detailed box plots (Fig 6A) or their medians were normalized on a 0–1 scale (setting fold differences >1 to 1/fold difference) and averaged over all ten antibody specificities (Fig 6B). For the next-generation NFP algorithms, an adjustment procedure was developed that incorporated the expected prediction accuracy for each of the ten antibody specificities for a given virus panel. The expected prediction accuracy *e*_*i*_ for a given antibody specificity *i* was determined by the true/false positive rate as determined on the test set of pairwise simulated sera (e.g., S8B Fig), which was independent from the simulated sera described in this section. Specifically, *e*_*i*_ was defined as the ratio *t*_*i*_*/(n*_*i*_*+t*_*i*_*)*, where *t*_*i*_ is the number of sera with (a) two predicted positive signals and (b) a positive signal for specificity *i*; and *n*_*i*_ is the number of false negative sera for specificity *i*. In effect, *e*_*i*_ allows us to estimate the false negative rate from the observed frequency of positive signals for specificity *i* in sera that are predicted to have two positive signals. To apply the adjustment procedure in prospective cohort-level analysis, the frequency *f*_*i*_ of a given antibody specificity *i*, for sera with two positive signals, was computed as *x*_*i*_*/e*_*i*_, where *x*_*i*_ is the number of sera with positive signals for specificity *i*. This frequency was then combined with the (unadjusted) frequencies for the remaining sera (for which the number of positive signals was different from two), in order to obtain the overall NFP-predicted frequency for a given antibody specificity.

### Large-scale analysis of HIV-infected donors

HIV-1 neutralization for a set of 205 donor plasma was obtained from published data, which also included information about the clade of infection [63, 76]. The 132 strains from the monoclonal antibody dataset described above were compared to the strains from the plasma dataset, and a common panel of 69 strains was selected (S8A Fig). Plasma samples with missing data for the 69 common strains were discarded from the analysis, resulting in a set of 189 samples. A virus panel search for optimized NFP accuracy was performed, and a panel of size 50 with reduced false positive rates was selected for the plasma analysis (S8A and S8B Fig). We specifically selected a panel with reduced false positives, in order to facilitate the estimation of specificities at a population level given the individual predictions (see section above). Samples with less than 30% neutralization breadth were discarded from further analysis (since the information content for sera with low neutralization breadth does not permit accurate delineation of the polyclonal signal), resulting in a final set of 143 plasma for analysis by the next-generation NFP algorithms. A residual score cutoff of greater than 0.085 (greater than the max for all simulated sera with known dominant specificities, for the selected 50-strain panel) was used for predicting plasma with potentially novel specificities. Cutoffs of less than or equal to -0.1 for residual scores, 0.06 for the frequency of random signals, and 0.065 for the median of the delineation scores were used for dividing the remaining plasma samples into samples predicted to have dominant known specificities and samples with inconclusive delineation.

### Statistical methods

GraphPad Prism, Octave, and Mathematica scripts were used for performing statistical analysis.

### Data deposition

Data deposited in the Dryad repository: http://dx.doi.org/10.5061/dryad.c73q1 [77]

## Supporting Information

S1 Fig Next-generation neutralization fingerprinting algorithms. (A) Schematic of the NFP approach. Polyclonal serum neutralization is represented as a combination of the neutralization fingerprints of a discrete set of epitope-specific antibody clusters. Neutralization data is displayed as a heatmap, with each colored box representing the neutralization potency against a single virus. (B) Comparison between algorithm features for the original vs. next-generation NFP algorithms. Features that are different from the original NFP and new features not in the original NFP are highlighted in green.
(PDF)

S2 Fig Neutralization fingerprinting analysis of monoclonal antibody combinations. (A) Neutralization fingerprinting analysis of experimental antibody combinations of 2, 3, and 4 broadly neutralizing antibodies. For each antibody combination, the NFP algorithm was applied to predict component antibody specificities. (B) Neutralization data and Spearman correlation coefficients for simulated and experimentally determined combinations of antibody pairs. For each antibody pair (bar), the experimental (left) and simulated (right) neutralization potency is shown for a set of 125 HIV-1 strains (rows), with heatmap colored according to potency (white for no neutralization; green-yellow-red for increasing potency). (C) Neutralization breadth of simulated sera.
(PDF)

S3 Fig Selection of 20-strain panel f61 and computational comparison to the published 21-strain panel. (A) Example of neutralization-based antibody clustering for two arbitrary virus panels. The clustering on top has a better agreement with known antibody epitopes than the clustering at the bottom, in which examples of misclassified antibodies are highlighted with arrows. (B) Neutralization breadth of simulated sera for panel f61. (C) (top) Names and clades for the strains from virus panel f61 (left) and the published 21-strain panel (right). (bottom) The respective strains are highlighted (red) on a phylogenetic tree for the set of 132 strains used in the panel search; for the published 21-strain panel, shown are the 17 strains that overlap with the 132-strain panel. (D) Detailed view of true positive (red) and false positive (blue) signals for each simulated serum for panel f61 (left) and the published 21-strain panel (right).
(PDF)

S4 Fig Correspondence between false negative and false positive specificities in simulated sera with known specificities for virus panel f61. For all simulated sera with false negative signal for a given specificity (rows), shown is the frequency of having a false positive signal for each of the other specificities (columns). Values in a row add up to 1. A larger frequency within a row could indicate that the given specificity is more likely to be erroneously substituted in the prediction by the specificity shown in the respective column (for example, a false negative for PGT151-like was almost exclusively associated with a false positive for b12-like signals).
(PDF)

S5 Fig Frequency of bNAb signals predicted in analysis of sera with unknown specificities for virus panel f61. Simulated sera with unknown specificities were grouped based on percent neutralization breadth (x-axis), and the fraction of sera (y-axis) with an NFP prediction for each of the reference bNAb specificities (color dots/lines) was calculated for each serum group. For each bNAb specificity, the fraction of sera with positive signals for that specificity was generally consistent for all serum groups.
(PDF)

S6 Fig Neutralization data for experimental monoclonal antibody combinations (IC_50_ values, top) and donor sera (ID_50_ values, bottom) against virus panel f61. The heatmap is colored according to potency (white for no neutralization; green-yellow-orange-red for increasing potency).
(PDF)

S7 Fig Confidence scores for eight donor sera with virus panel f61. Prediction confidence was calculated for the sera presented in Fig 4E. (A) Residual scores for the eight donor sera (dotted lines, labeled) are plotted against the scores for simulated sera with dominant known vs. unknown antibody specificities. (B) Frequency of random signals (y-axis) vs. median of delineation scores (x-axis) for the set of simulated sera with dominant known specificities (black dots) and the eight donor sera (red dots). With the exception of CAP256, CAP255, and C38, the dots for the other donor sera overlap near the origin. (C) Table with serum confidence scores from (A,B), and epitope specificity of antibodies isolated from the donors, with corresponding references. Higher values for the confidence scores are associated with lower confidence in the NFP predictions for a given serum.
(PDF)

S8 Fig Algorithm for and application of population-level NFP analysis. (A) Phylogenetic tree of the full 132-strain panel, highlighting the 69 strains (green) in common with the neutralization data for the large-scale HIV-infected donor analysis, as well as the selected 50-strain panel (red) for NFP analysis. (B) True positive (red) and false positive (blue) signals on simulated sera with the selected 50-strain panel. (C) Delineation and confidence scores for plasma characterized as inconclusive (grey labels) or with a predicted dominance of potentially novel specificities (orange labels). The delineation scores are shown only for reference here since they were classified as less or not reliable compared to the 50 samples with predicted dominant known specificities.
(PDF)

S9 Fig Neutralization fingerprint-based antibody clustering for the extended set of antibodies used for generation of the test set of simulated sera. Data from multiple repeats of the neutralization experiments for some antibodies were included as separate datapoints. Antibodies are colored according to their epitope specificity.
(PDF)

S10 Fig Serum delineation accuracy of top 5,000 virus panels of size 20 on a test set of sera that does (x-axis) or does not (y-axis) include bNAb clonal relatives. (PDF)
