# Supplementary figures and images for: Mapping Polyclonal HIV-1 Antibody Responses via Next-Generation Neutralization Fingerprinting
Source: PLoS Pathog. 2017 Jan 4;13(1):e1006148. doi: 10.1371/journal.ppat.1006148 (PMC5241146; doi:10.1371/journal.ppat.1006148)

Figure S1

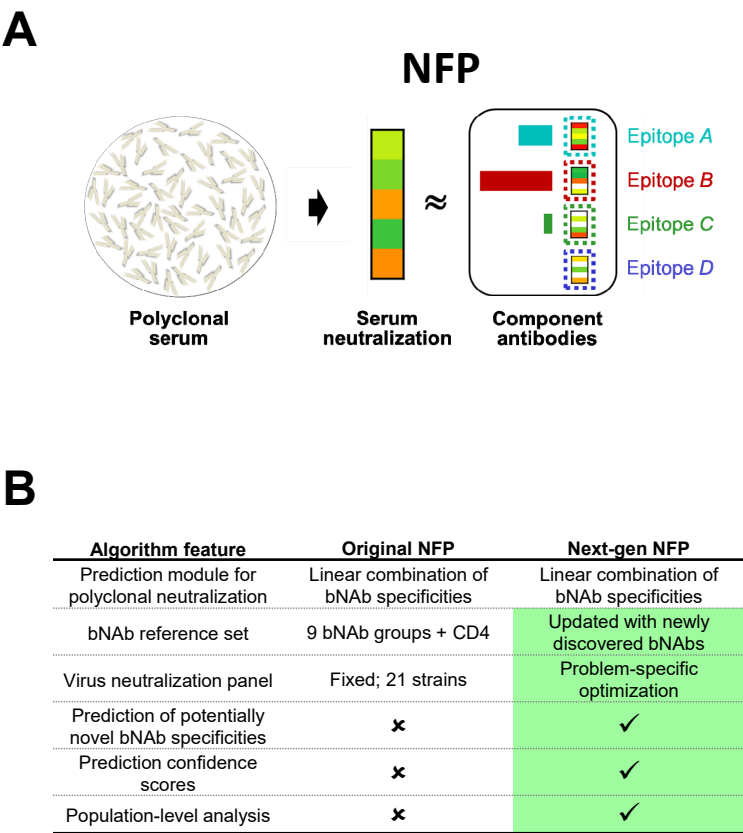

Supplement: S1 Fig — (A) Schematic of the NFP approach. Polyclonal serum neutralization is represented as a combination of the neutralization fingerprints of a discrete set of epitope-specific antibody clusters. Neutralization data is displayed as a heatmap, with each colored box representing the neutralization potency against a single virus. (B) Comparison between algorithm features for the original vs. next-generation NFP algorithms. Features that are different from the original NFP and new features not in the original NFP are highlighted in green. (PDF) [file ppat.1006148.s001.pdf]

Figure S2

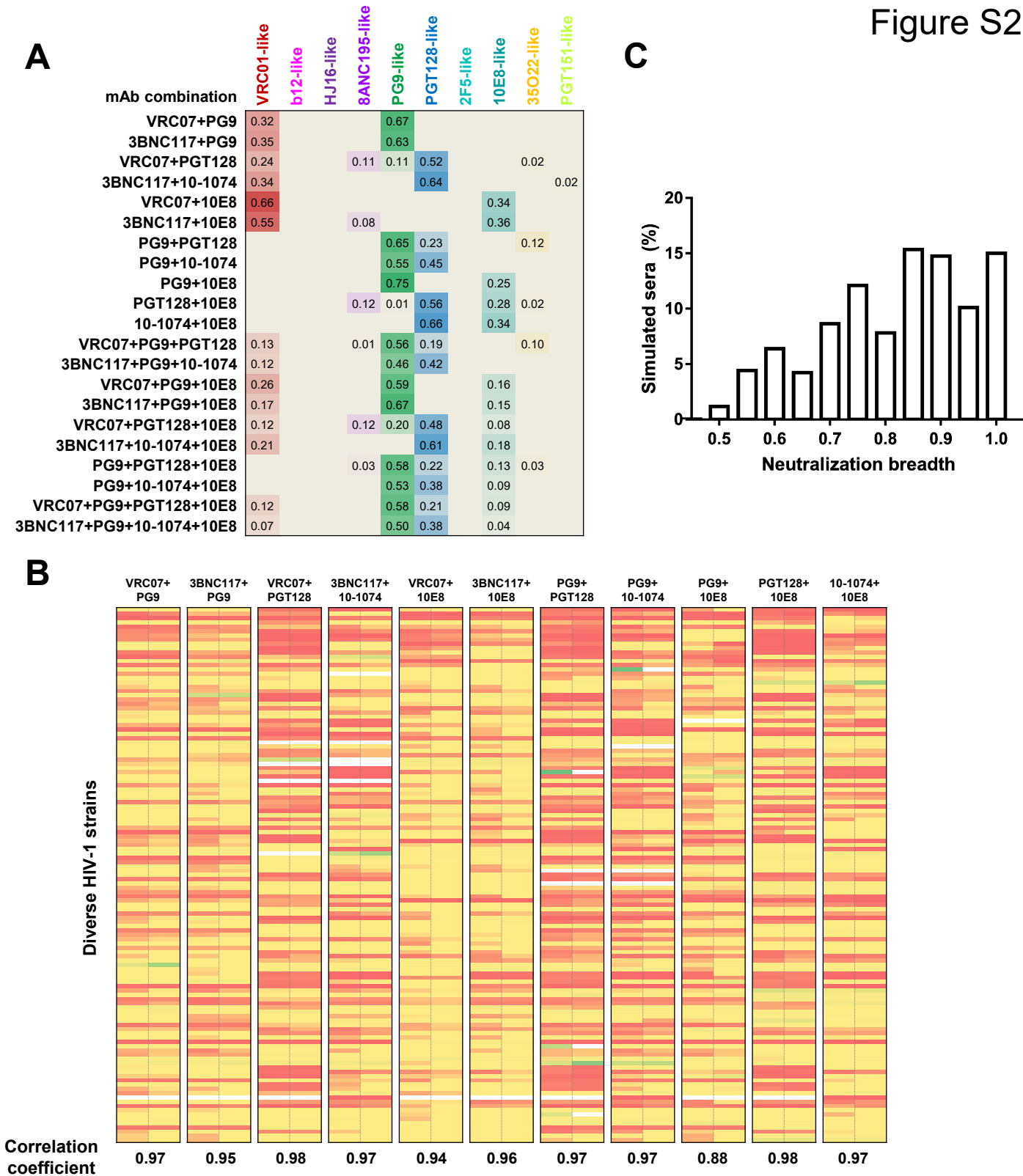

Supplement: S2 Fig — (A) Neutralization fingerprinting analysis of experimental antibody combinations of 2, 3, and 4 broadly neutralizing antibodies. For each antibody combination, the NFP algorithm was applied to predict component antibody specificities. (B) Neutralization data and Spearman correlation coefficients for simulated and experimentally determined combinations of antibody pairs. For each antibody pair (bar), the experimental (left) and simulated (right) neutralization potency is shown for a set of 125 HIV-1 strains (rows), with heatmap colored according to potency (white for no neutralization; green-yellow-red for increasing potency). (C) Neutralization breadth of simulated sera. (PDF) [file ppat.1006148.s002.pdf]

Figure S3

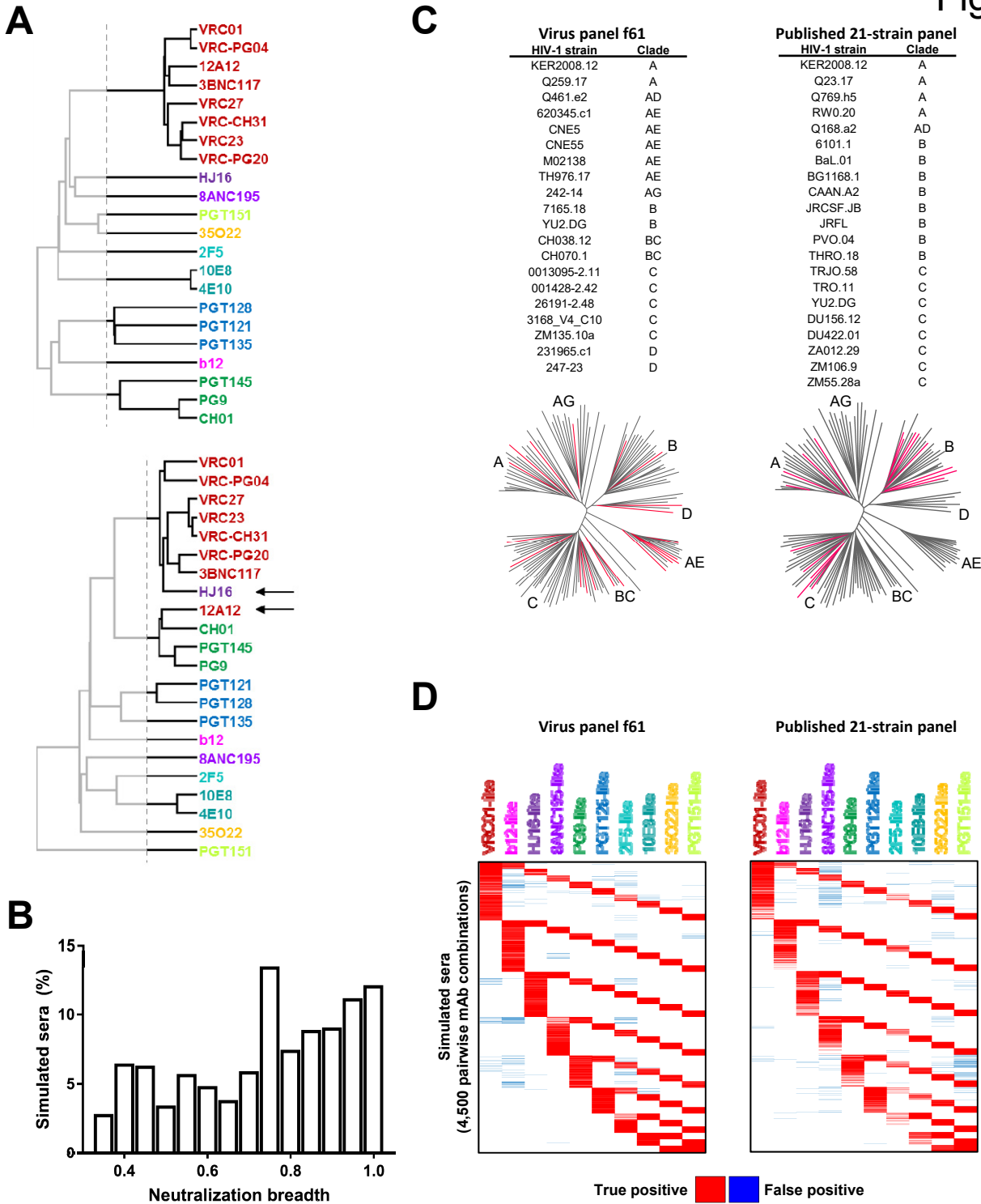

Supplement: S3 Fig — (A) Example of neutralization-based antibody clustering for two arbitrary virus panels. The clustering on top has a better agreement with known antibody epitopes than the clustering at the bottom, in which examples of misclassified antibodies are highlighted with arrows. (B) Neutralization breadth of simulated sera for panel f61. (C) (top) Names and clades for the strains from virus panel f61 (left) and the published 21-strain panel (right). (bottom) The respective strains are highlighted (red) on a phylogenetic tree for the set of 132 strains used in the panel search; for the published 21-strain panel, shown are the 17 strains that overlap with the 132-strain panel. (D) Detailed view of true positive (red) and false positive (blue) signals for each simulated serum for panel f61 (left) and the published 21-strain panel (right). (PDF) [file ppat.1006148.s003.pdf]

Figure S5

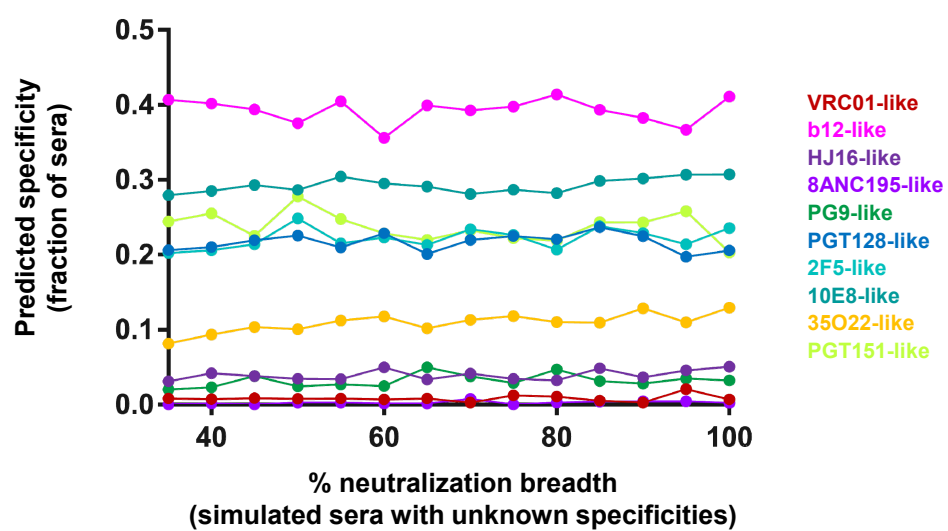

Supplement: S5 Fig — Simulated sera with unknown specificities were grouped based on percent neutralization breadth (x-axis), and the fraction of sera (y-axis) with an NFP prediction for each of the reference bNAb specificities (color dots/lines) was calculated for each serum group. For each bNAb specificity, the fraction of sera with positive signals for that specificity was generally consistent for all serum groups. (PDF) [file ppat.1006148.s005.pdf]

Figure S8

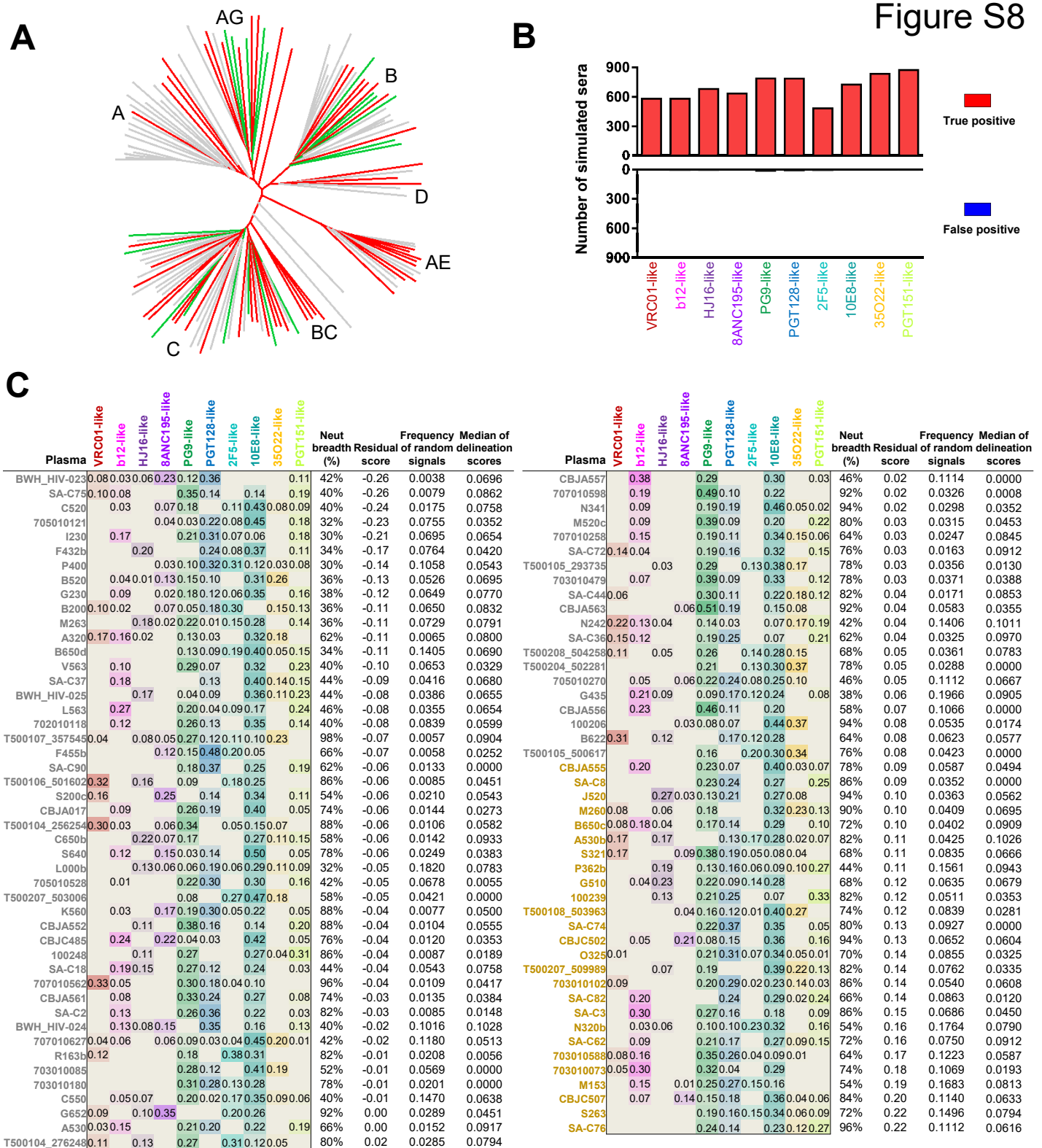

Supplement: S8 Fig — (A) Phylogenetic tree of the full 132-strain panel, highlighting the 69 strains (green) in common with the neutralization data for the large-scale HIV-infected donor analysis, as well as the selected 50-strain panel (red) for NFP analysis. (B) True positive (red) and false positive (blue) signals on simulated sera with the selected 50-strain panel. (C) Delineation and confidence scores for plasma characterized as inconclusive (grey labels) or with a predicted dominance of potentially novel specificities (orange labels). The delineation scores are shown only for reference here since they were classified as less or not reliable compared to the 50 samples with predicted dominant known specificities. (PDF) [file ppat.1006148.s008.pdf]

Figure S9

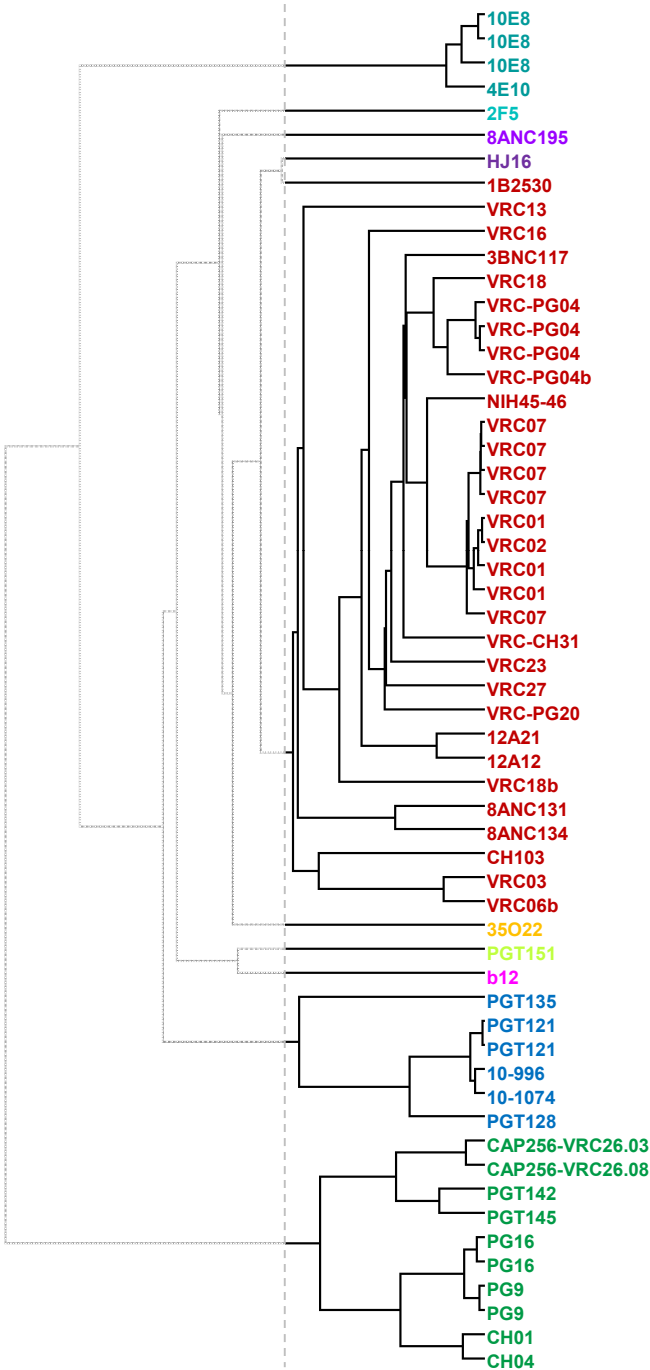

Supplement: S9 Fig — Data from multiple repeats of the neutralization experiments for some antibodies were included as separate datapoints. Antibodies are colored according to their epitope specificity. (PDF) [file ppat.1006148.s009.pdf]

Figure S10

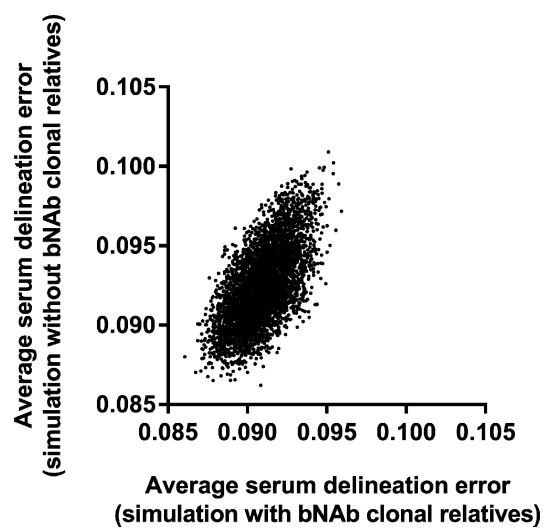

Supplement: S10 Fig — (PDF) [file ppat.1006148.s010.pdf]
