# Supplementary material for: Mapping Polyclonal HIV-1 Antibody Responses via Next-Generation Neutralization Fingerprinting
Source: PLoS Pathog. 2017 Jan 4;13(1):e1006148. doi: 10.1371/journal.ppat.1006148 (PMC5241146; doi:10.1371/journal.ppat.1006148)
Supplement: S4 Fig — For all simulated sera with false negative signal for a given specificity (rows), shown is the frequency of having a false positive signal for each of the other specificities (columns). Values in a row add up to 1. A larger frequency within a row could indicate that the given specificity is more likely to be erroneously substituted in the prediction by the specificity shown in the respective column (for example, a false negative for PGT151-like was almost exclusively associated with a false positive for b12-like signals). (PDF) [file ppat.1006148.s004.pdf]

# Figure S4

**False positive specificity**

| False negative specificity | VRC01-like | b12-like | HJ16-like | 8ANC195-like | PG9-like | PGT128-like | 2F5-like | 10E8-like | 35O22-like | PGT151-like |
|---|---|---|---|---|---|---|---|---|---|---|
| VRC01-like | | 0.4412 | 0.1176 | 0.0588 | 0 | 0.0294 | 0.2647 | 0.0588 | 0 | 0.0294 |
| b12-like | 0 | | 0.0625 | 0.5 | 0 | 0.0625 | 0 | 0 | 0 | 0.375 |
| HJ16-like | 0.4737 | 0.3158 | | 0 | 0 | 0 | 0.2105 | 0 | 0 | 0 |
| 8ANC195-like | 0.1129 | 0.5161 | 0.0161 | | 0 | 0.0645 | 0.2903 | 0 | 0 | 0 |
| PG9-like | 0.1379 | 0.5402 | 0.0345 | 0 | | 0 | 0.2874 | 0 | 0 | 0 |
| PGT128-like | 0.25 | 0.5 | 0 | 0 | 0 | | 0.25 | 0 | 0 | 0 |
| 2F5-like | 0.15 | 0 | 0.05 | 0 | 0 | 0.05 | | 0.6 | 0.05 | 0.1 |
| 10E8-like | 0.3 | 0.3 | 0 | 0 | 0 | 0.3 | 0.1 | | 0 | 0 |
| 35O22-like | 0 | 0.4 | 0.2 | 0 | 0 | 0 | 0.4 | 0 | | 0 |
| PGT151-like | 0 | 0.8125 | 0 | 0 | 0 | 0 | 0.1875 | 0 | 0 | |
