# Supplementary material for: Mapping Polyclonal HIV-1 Antibody Responses via Next-Generation Neutralization Fingerprinting
Source: PLoS Pathog. 2017 Jan 4;13(1):e1006148. doi: 10.1371/journal.ppat.1006148 (PMC5241146; doi:10.1371/journal.ppat.1006148)
Supplement: S6 Fig — The heatmap is colored according to potency (white for no neutralization; green-yellow-orange-red for increasing potency). (PDF) [file ppat.1006148.s006.pdf]

Figure S6

IC_{50}

strain	50%HJ16, 50%VRC01	50%PG9, 50%VRC01	50%8ANC195, 50%b12	50%10E8, 50%b12	50%HJ16, 50%PGT128	50%8ANC195, 50%2F5	50%PG9, 50%PGT151	50%35O22, 50%PGT128	50%10E8, 50%2F5	50%35O22, 50%PGT151	66%VRC01, 33%10E8	33%VRC01, 66%10E8	66%b12, 33%2F5	33%b12, 66%2F5	66%HJ16, 33%35O22	33%HJ16, 66%35O22	66%PG9, 33%8ANC195	33%PG9, 66%8ANC195	66%PGT128, 33%PGT151	33%PGT128, 66%PGT151
7165.18	>50	>50	0.328	0.627	0.029	0.113	0.092	0.019	0.295	0.051	0.440	0.338	3.84	1.30	>50	>50	0.403	0.186	0.009	0.019
0013095-2.11	0.339	0.055	0.796	0.076	>50	0.616	0.033	>50	0.056	>50	0.046	0.042	>50	>50	>50	>50	0.148	0.145	0.113	0.093
001428-2.42	0.054	0.010	>50	3.61	0.076	>50	0.012	0.042	2.40	0.036	0.024	0.068	>50	>50	>50	>50	0.006	0.016	0.020	0.030
231965.c1	0.914	0.325	0.130	0.224	>50	0.778	0.025	>50	3.77	0.012	0.496	1.40	0.143	0.208	>50	>50	0.269	0.541	0.048	0.017
242-14	>50	0.087	4.46	1.41	>50	0.565	0.027	>50	0.524	0.020	0.804	0.596	1.63	0.591	>50	>50	0.065	0.150	0.048	0.028
247-23	12.8	0.315	>50	0.306	>50	3.44	0.331	0.006	0.300	0.005	0.395	0.271	4.73	1.61	0.019	0.007	0.319	0.859	>50	>50
26191-2.48	0.056	0.213	4.91	1.07	0.021	>50	0.305	0.048	2.58	>50	0.255	0.418	2.56	5.08	0.043	0.091	0.172	0.286	0.022	0.031
3168_V4_C10	0.521	0.201	16.9	2.95	>50	12.1	0.329	0.020	3.00	0.018	0.155	0.355	>50	19.9	0.019	0.013	0.174	0.316	1.60	2.54
620345.c1	>50	0.576	>50	0.746	>50	1.56	0.100	>50	0.413	>50	1.03	0.543	4.38	1.00	>50	>50	0.665	>50	>50	>50
CH038.12	1.17	0.376	0.346	0.274	0.014	6.65	1.41	0.010	0.728	>50	0.289	0.340	0.205	0.639	7.65	>50	0.443	0.714	0.010	0.025
CH070.1	>50	0.036	>50	5.50	0.096	>50	0.034	0.067	6.12	>50	2.07	3.44	>50	>50	>50	>50	0.022	0.064	0.034	0.112
CNE5	0.134	0.023	1.05	0.891	0.056	1.66	0.031	0.049	1.77	>50	0.312	0.390	4.78	3.12	0.070	0.069	0.015	0.031	0.062	0.095
CNE55	0.380	0.056	1.13	0.113	>50	0.181	0.144	0.277	0.085	>50	0.110	0.104	4.43	0.436	>50	>50	0.146	0.293	>50	>50
KER2008.12	1.43	0.038	4.33	>50	>50	3.01	0.018	0.005	23.5	0.004	0.711	1.29	>50	9.70	0.005	0.002	0.019	0.032	0.032	0.016
M02138	1.27	0.306	4.95	0.044	>50	0.332	0.654	>50	0.050	>50	0.058	0.029	0.175	0.093	42.5	>50	0.280	0.378	>50	>50
Q259.17	0.153	0.065	>50	7.33	>50	38.3	0.074	>50	5.72	0.051	0.070	0.188	45.6	16.7	0.121	0.248	0.052	0.141	0.635	0.934
Q461.e2	0.192	0.705	2.44	2.59	0.312	1.89	0.265	0.005	2.40	0.004	0.532	0.632	31.9	17.5	0.004	0.003	1.05	0.855	0.281	0.227
TH976.17	0.053	0.332	0.775	0.709	0.088	0.256	>50	>50	0.295	>50	0.141	0.084	5.21	1.35	0.027	0.069	1.07	0.616	>50	>50
YU2.DG	0.195	0.296	1.21	1.70	0.106	2.08	1.26	0.058	2.94	0.203	0.190	0.285	2.31	3.36	0.395	0.529	0.700	0.718	0.084	0.162
ZM135.10a	1.67	1.62	>50	0.181	>50	>50	>50	10.3	0.200	>50	0.352	0.192	>50	>50	>50	>50	>50	>50	>50	>50

ID_{50}

strain	NIAID 45	Z258	Rhesus CE8J	CAP206	CAP255	C38	CAP256	CHAVI 0219
7165.18	79	64	259	25	136	112	<20	96
0013095-2.11	715	709	<20	763	22	563	606	220
001428-2.42	2466	1021	167	68	121	1217	191	1005
231965.c1	186	160	<20	<20	738	174	<20	70
242-14	221	302	<20	160	<20	449	3726	304
247-23	73	53	<20	37	<20	117	1137	145
26191-2.48	126	152	69	<20	60	319	3418	173
3168_V4_C10	279	114	<20	<20	<20	103	27	201
620345.c1	<20	<20	<20	62	21	<20	7744	165
CH038.12	177	94	926	40	520	212	2331	101
CH070.1	76	<20	1283	<20	527	21	4774	322
CNE5	121	105	<20	<20	22	72	2724	377
CNE55	284	435	<20	66	45	92	11022	953
KER2008.12	163	147	<20	<20	35	550	282	375
M02138	166	232	59	266	<20	156	75	141
Q259.17	1736	344	<20	26	<20	411	6297	1016
Q461.e2	86	62	<20	<20	21	65	221	150
TH976.17	339	437	<20	113	25	150	1462	1238
YU2.DG	767	224	205	24	36	483	<20	976
ZM135.10a	103	154	21	64	21	27	116	57
