# Supplementary material for: Mapping Polyclonal HIV-1 Antibody Responses via Next-Generation Neutralization Fingerprinting
Source: PLoS Pathog. 2017 Jan 4;13(1):e1006148. doi: 10.1371/journal.ppat.1006148 (PMC5241146; doi:10.1371/journal.ppat.1006148)
Supplement: S7 Fig — Prediction confidence was calculated for the sera presented in Fig 4E. (A) Residual scores for the eight donor sera (dotted lines, labeled) are plotted against the scores for simulated sera with dominant known vs. unknown antibody specificities. (B) Frequency of random signals (y-axis) vs. median of delineation scores (x-axis) for the set of simulated sera with dominant known specificities (black dots) and the eight donor sera (red dots). With the exception of CAP256, CAP255, and C38, the dots for the other donor sera overlap near the origin. (C) Table with serum confidence scores from (A,B), and epitope specificity of antibodies isolated from the donors, with corresponding references. Higher values for the confidence scores are associated with lower confidence in the NFP predictions for a given serum. (PDF) [file ppat.1006148.s007.pdf]

Figure S7

A

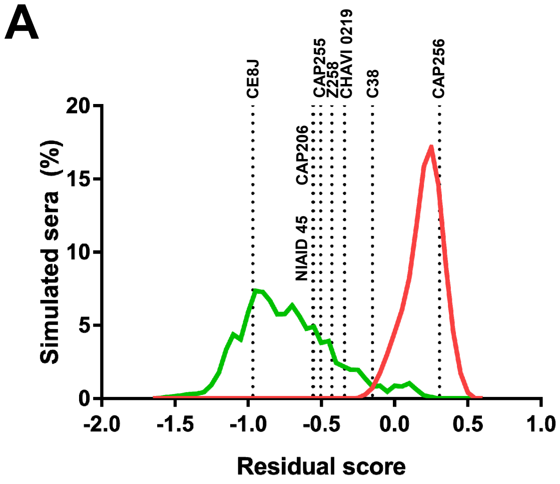

B

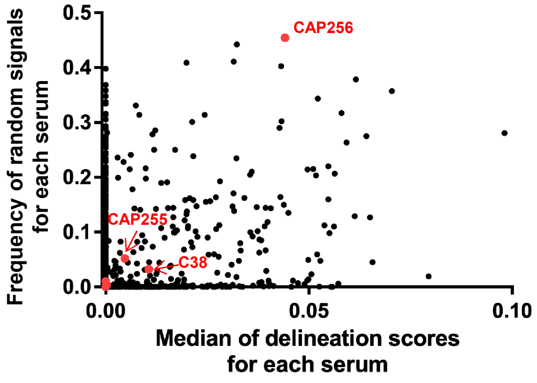

C

Donor	Residual score	Median of delineation scores	Frequency of random signals	Isolated bNAbs (specificity)	References
**NIAID 45**	-0.56	0.0000	0.0103	VRC01 (VRC01-like)	(9)
**Z258**	-0.43	0.0000	0.0103	VRC27 (VRC01-like)	(68)
**Rhesus CE8J**	-0.97	0.0000	0.0004	none (plasma mapped to PGT128-like)	(67)
**CAP206**	-0.55	0.0003	0.0032	CAP2016-CH12 (10E8-like)	(69)
**CAP255**	-0.50	0.0047	0.0519	epitope mapping to N332	(37)
**C38**	-0.15	0.0106	0.0324	VRC16, VRC18 (VRC01-like)	(68)
**CAP256**	0.31	0.0441	0.4544	CAP256-VRC26 (PG9-like)	(14)
**CHAVI 0219**	-0.34	0.0000	0.0107	CH01, VRC-CH31 (PG9-like, VRC01-like)	(18)
